# Genotoxic effects of base and prime editing in human hematopoietic stem cells

**DOI:** 10.1038/s41587-023-01915-4

**Published:** 2023-09-07

**Authors:** Martina Fiumara, Samuele Ferrari, Attya Omer-Javed, Stefano Beretta, Luisa Albano, Daniele Canarutto, Angelica Varesi, Chiara Gaddoni, Chiara Brombin, Federica Cugnata, Erika Zonari, Matteo Maria Naldini, Matteo Barcella, Bernhard Gentner, Ivan Merelli, Luigi Naldini

**Affiliations:** 1 San Raffaele Telethon Institute for Gene Therapy, IRCCS San Raffaele Scientific Institute, Milan, Italy; 2 Vita-Salute San Raffaele University, Milan, Italy; 3 Pediatric Immunohematology Unit and BMT Program, IRCCS San Raffaele Scientific Institute, Milan, Italy; 4 University Center for Statistics in the Biomedical Sciences, Vita-Salute San Raffaele University, Milan, Italy; 5 National Research Council, Institute for Biomedical Technologies, Segrate, Italy

**Keywords:** Targeted gene repair, Stem-cell biotechnology, Haematopoietic stem cells

## Abstract

Base and prime editors (BEs and PEs) may provide more precise genetic engineering than nuclease-based approaches because they bypass the dependence on DNA double-strand breaks. However, little is known about their cellular responses and genotoxicity. Here, we compared state-of-the-art BEs and PEs and Cas9 in human hematopoietic stem and progenitor cells with respect to editing efficiency, cytotoxicity, transcriptomic changes and on-target and genome-wide genotoxicity. BEs and PEs induced detrimental transcriptional responses that reduced editing efficiency and hematopoietic repopulation in xenotransplants and also generated DNA double-strand breaks and genotoxic byproducts, including deletions and translocations, at a lower frequency than Cas9. These effects were strongest for cytidine BEs due to suboptimal inhibition of base excision repair and were mitigated by tailoring delivery timing and editor expression through optimized mRNA design. However, BEs altered the mutational landscape of hematopoietic stem and progenitor cells across the genome by increasing the load and relative proportions of nucleotide variants. These findings raise concerns about the genotoxicity of BEs and PEs and warrant further investigation in view of their clinical application.

## Abstract (web summary)

Base and prime editors induce double-strand breaks, deletions and translocations in hematopoietic cells.

## Main

Gene editing represents a promising tool to engineer human hematopoietic stem/progenitor cells (HSPCs), opening the possibility to precisely correct disease-causing mutations while limiting the risk of genome-wide insertional mutagenesis and unregulated expression of the transgene^1^. In the last decade, CRISPR–Cas-engineered nucleases coupled with a single guide RNA (gRNA) have been widely used to introduce site-specific DNA double-strand breaks (DSBs)^2,3^. The DSBs generated can be repaired either by (1) non-homologous/microhomology-mediated end joining, often leading to insertion or deletion (indels) of some nucleotides at the break site and resulting in disruption of coding or regulatory function, or (2) homology-directed recombination (HDR), which exploits an exogenous DNA repair template with homologous sequences, resulting in gene replacement or insertion^4^. However, several biological barriers constrain efficiency and tolerability of nuclease-dependent gene editing in HSPCs. Nuclease-induced DSBs at on- and off-target sites trigger the activation of a p53-dependent DNA damage response^5,6^, and their processing may sometimes lead to large deletions, translocations and chromothripsis, thus enhancing genotoxic risk^7–11^. Moreover, HDR-based editing is confined to S/G2 cell cycle phases, limiting it to the most primitive and quiescent compartment of HSPCs^12^.

Recently, Cas nickase (nCas)-based technologies, such as base editing and prime editing, opened the possibility to generate small edits while bypassing DNA DSB and HDR engagement^13^ and ensuring a more homogeneous genetic outcome at the target sites^14^. Base editors (BEs) consist of a nucleotide deamination domain, mainly acting on single-stranded DNA with different base specificity and origin, and an nCas, which introduces a single-strand break (SSB) on the gRNA targeted strand to favor establishment of the introduced mutation over repair of the base mismatch. Depending on specificity and outcome, BEs are classified as cytidine BEs (CBEs; enabling the transition of C•G to T•A) and adenine BEs (ABEs; enabling the transition of A•T to G•C)^15^. Different types of deaminases have been coupled with nCas orthologs and engineered to tailor the editing window and broaden genome accessibility^13^. Whereas deaminated cytidine (uracil) is rapidly recognized by uracil glycosylase (UG) and restored by base excision repair (BER), deaminated adenosine (inosine) is not recognized by the eukaryotic DNA repair machinery. Thus, CBEs were further engineered by adding one or two UG inhibitor (UGI) domains from a bacteriophage protein, improving their efficiency^16^. BEs have been successfully tested in different cell types, including primary HSPCs, with encouraging results in terms of efficiency and persistence of edited cells^17–21^, and an ABE is in clinical testing for the treatment of sickle cell disease (NCT05456880).

Whereas base editing is mostly confined to generating single-base transitions, prime editing allows for the generation of single-nucleotide variants (SNVs) of all types as well as small intended indels. Prime editor 2 (PE2) is comprised of an nCas9 fused with a reverse transcriptase (RT) domain from the Moloney murine leukemia virus. PE2 is coupled to a prime editor gRNA (pegRNA) to induce an nCas9-mediated DNA SSB; the pegRNA also provides the template for reverse transcription originating from the cut strand. To improve efficiency, PE2 can be paired with an additional gRNA specific to the non-edited strand, forcing the use of the edited strand to resolve the heteroduplex (PE3)^22^. Furthermore, other PE and pegRNA versions were recently reported to increase editing efficiency^23–26^, albeit remaining lower than what can be achieved using other editing systems.

Despite their promise for less harmful and more precise genetic engineering, little is known about the short- and long-term toxicity of BEs and PEs in human cells. Concerns include conversion of the DNA SSB into a DSB during cell replication, expression of constitutive deaminases/RT that may have gRNA-independent genome-wide mutagenic potential and adverse cellular responses triggered by the reagents and nucleic acid processing intermediates of base and prime editing. Here, we perform a comparative assessment of state-of-the-art BEs and PEs versus Cas9 editing in HSPCs in terms of efficiency, cytotoxicity, detrimental cellular responses and on-target and genome-wide genotoxicity. These analyses uncovered both advantages and limitations of the emerging nCas-based systems. Although optimized timing and extent of editor expression allowed reaching the intended base or prime editing with high efficiency in long-term repopulating HSPCs, we uncovered detrimental innate sensing of these artificial molecules and their activity, which could be overcome only in part, and a lowered but not abrogated induction of DNA DSBs and their genotoxic byproducts. Moreover, our efforts to perform unbiased genome-wide analyses to capture gRNA-independent mutagenic events uncovered BE-induced perturbations of the mutational landscape of treated HSPCs.

## Results

### Base editing leads to imprecise outcomes at target sites ascribed to DNA DSBs

**Fig. 1:**
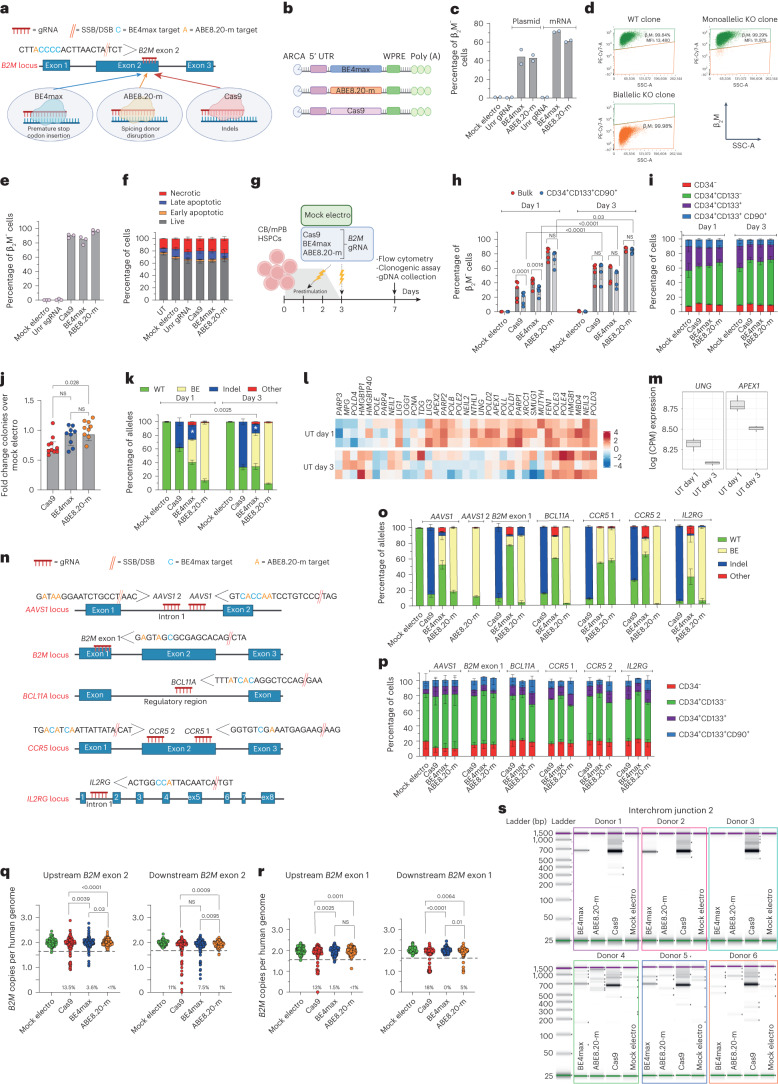
Base editing of human HSPCs results in imprecise outcomes, including large deletions and translocations. **a**,**b**, Schematic representation of the *B2M* exon 2 editing strategies and their cognate genetic outcomes (**a**) and the editor mRNAs (**b**); ARCA, anti-reverse cap analog; WPRE, woodchuck hepatitis virus post-transcriptional regulatory element. **c**, Percentage of β_2_M^−^ B lymphoblastoid cells as measured by flow cytometry (*n* = 2). Data are shown as median values; Unr, unrelated; Mock electro, mock electroporated. **d**, Flow cytometry plots of three representative B lymphoblastoid clones showing wild-type (WT), monoallelic and biallelic editing confirmed by Sanger sequencing; SSC-A, side scatter; MFI, mean fluorescence intensity. **e**, Percentage of β_2_M^−^ human T cells 7 d after treatments (*n* = 3). Data are shown as median values. **f**, Percentage of live, early/late apoptotic and necrotic T cells 24 h after treatments; UT, untreated (*n* = 3). Data are shown as mean ± s.e.m. **g**, Experimental workflow for *B2M* editing in CB or mPB HSPCs. **h**, Percentage of β_2_M^−^ CB HSPCs edited at day 1 or day 3 after thawing (*n* = 5). Data are shown as median values with interquartile range (IQR) and were analyzed by a linear mixed effects (LME) model followed by post hoc analysis; NS, not significant. **i**, Proportion of cellular subpopulations within CB HSPCs from experiments in **h** (*n* = 5). Data are shown as mean ± s.e.m. and were analyzed by an LME model followed by post hoc analysis. **j**, Fold change in the number of colonies generated by CB or mPB HPSCs over mock electroporation (*n* = 10). Data are shown as median values with IQR and were analyzed by Kruskal–Wallis test with Dunn’s multiple comparison test. **k**, Percentage of *B2M* alleles measured by deep sequencing (WT or carrying the described editing outcomes in CB HSPCs; *n* = 5 for day 1; *n* = 6, 7, 7 and 7 for day 3). Data are shown as mean ± s.e.m. and were analyzed by Mann–Whitney test. Statistics is denoted by asterisks. **l**, Heat map of normalized read counts for genes belonging to the BER pathway (KEGG database hsa03410) in untreated CB HSPCs cultured for 1 or 3 d (*n* = 3). **m**, *UNG* and *APEX1* log counts per million (CPM) reads in untreated CB HSPCs cultured for 1 or 3 d (*n* = 3). The center of the box plot represents the median, and boundaries represent first and third quartiles. Upper and lower whiskers extend 1.5× IQR from the hinge. **n**, Schematic representation of the *AAVS1*, *B2M* exon 1, *BCL11A*, *CCR5* and *IL2RG* editing strategies. **o**, Percentage of *AAVS1*, *B2M* exon 1, *BCL11A*, *CCR5* and *IL2RG* alleles measured by deep sequencing (WT or carrying the described editing outcomes in mPB HSPCs; *n* = 3 for *AAVS1* Cas9; *n* = 7 for *AAVS1* BE4max and ABE8.20-m; *n* = 3 for *B2M* exon 1, *BCL11A*, *CCR5* and *IL2RG*). Data are shown as mean ± s.e.m. **p**, Proportion of cellular subpopulations within mPB HSPCs from experiments in **o** (*n* = 3). Data are shown as mean ± s.e.m. Samples edited in *BCL11A*, *CCR5* and *IL2RG* were unified for statistical analysis using a Friedman test with Dunn’s multiple comparison on the most primitive compartments (CD34^+^CD133^+^ and CD34^+^CD133^+^CD90^+^), as experiments were performed in parallel on the same mPB HSPC donors. Cas9 and BE4max showed a significant reduction in the proportion of primitive compartments compared to ABE8.20-m (*P* = 0.016 and *P* < 0.0001, respectively). **q**, Copies of *B2M* sequences per human genome flanking the exon 2 target site in individual colonies generated by edited mPB HSPCs (*n* = 105, 188, 188 and 187 for the ‘upstream’ assay; *n* = 93, 188, 188 and 187 for the ‘downstream’ assay). Dashed lines indicate the lower limit of the confidence interval from mock-electroporated colonies. Data are shown as median values and were analyzed by Fisher’s exact test. **r**, Copies of *B2M* sequences per human genome flanking the exon 1 target site in individual colonies generated by edited mPB HSPCs (*n* = 89, 129, 130 and 125 for the ‘upstream’ assay; *n* = 89, 129, 129 and 126 for the ‘downstream’ assay). Dashed lines indicate the lower limit of the confidence interval from mock-electroporated colonies. Data are shown as median values and were analyzed by Fisher’s exact test. **s**, Images of capillary electropherograms showing amplification of interchromosomal (interchrom) junction 2 shown in Extended Data Fig. 1l after HSPC editing with two gRNAs targeting *B2M* exon 2 and *AAVS1* in six mPB donors. All statistical tests are two tailed. *n* indicates biologically independent experiments except for **q** and **r**, in which *n* indicates independent samples. Source data

**Extended Data Fig. 1:**
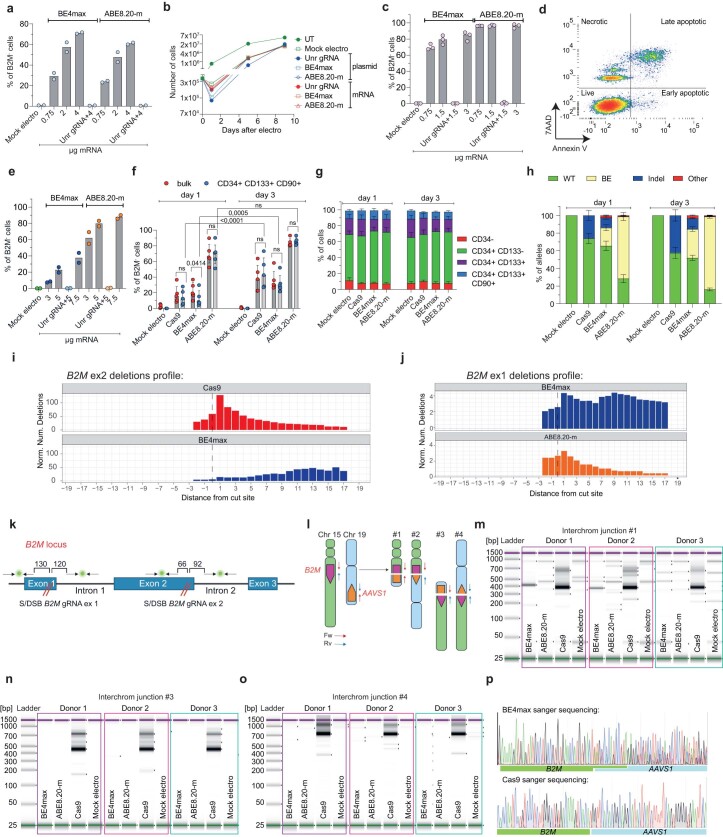
Efficient base editing in human HSPCs and repair outcomes at the target site. **a**, Percentage of B2M^−^ B-lymphoblastoid cells measured by flow cytometry 7 days after editing with different mRNA doses (n = 2). Unr: unrelated. Median. **b**, Growth curve of B-lymphoblastoid cells after treatments (n = 2). Median. **c**, Percentage of B2M^−^ T cells after editing with different mRNA doses (n = 3). Median. **d**, Representative plot showing gating strategy for live, early/late apoptotic and necrotic T cells. **e**, Percentage of B2M^−^ CB HSPCs after editing with different mRNA doses (n = 2). Median. **f**, Percentage of B2M^−^ mPB HSPCs edited at day 1 or day 3 post-thawing (n = 5). Median with IQR. LME followed by post hoc analysis. **g**, Proportion of cellular subpopulations within mPB HSPCs from experiments in ‘f’ (n = 5). Mean $\pm$ s.e.m. LME followed by post hoc analysis. **h**, Percentage of *B2M* alleles, measured by deep sequencing analysis, being WT or carrying the described editing outcomes in mPB HSPCs (n = 4,5,5,5 for day 1; n = 3,4,4,4 for day 3). Mean $\pm$ s.e.m. **i**, Distribution of the distance of indels from the *B2M* exon 2 S/DSB cut site in Cas9-edited (top) and BE4max-edited samples (bottom). **j**, Distribution of the distance of indels from the *B2M* exon 1 S/DSB cut site in BE4max-edited (top) and ABE8.20-m-edited samples (bottom). **k**, Schematic representation of the probes used for deletions detection at *B2M* target sites in exon 2 and exon 1. The distances between the target site and the closest primer of the ddPCR amplicons are shown. **l**, Schematic representation of translocations expected upon multiplexed *B2M* and *AAVS1* targeting. **m-o**, Representative capillary electropherogram showing amplification of #1 (m), #3 (n) and #4- (o) interchromosomal junctions upon HSPC editing with two gRNAs targeting *B2M* exon 2 and *AAVS1* (n = 3). **p**, Representative Sanger sequencing plot of *B2M* exon 2-*AAVS1* junction in samples from Fig. 1s. All statistical tests are two-tailed. n indicate biologically independent experiments.

To compare different editors side by side, we selected a state-of-the-art version of a CBE (BE4max)^27^ and an ABE (ABE8.20-m)^20^ and used a gRNA targeting the gene encoding β_2_-microglobulin (β_2_M)^20^ that can be coupled with either a BE or Cas9 to induce its knockout (KO). Because β_2_M is ubiquitously expressed on the cell surface, its KO allows straightforward quantification of editing efficiency by measuring lack of β_2_M expression via flow cytometry. Specifically, to induce *B2M* KO, Cas9 and BE4max introduce indels or a premature stop codon, respectively, while ABE8.20-m disrupts a splicing donor site (Fig. 1a). *B2M* editing by BE4max and ABE8.20-m in a B lymphoblastoid cell line reached around 60% *B2M* KO after electroporation of in vitro transcribed mRNAs in a dose-dependent manner (Fig. 1b,c and Extended Data Fig. 1a,b). As expected, *B2M* KO was not detected after BE combination with an unrelated gRNA. Flow cytometry and molecular analysis of single-cell-derived clones revealed that only biallelic KO reduced β_2_M expression on the cell surface (Fig. 1d). In human primary T cells, BE4max and Cas9 resulted in 80–90% *B2M*-KO cells, while ABE8.20-m reached >95% (Fig. 1e). All editing treatments showed comparable acute toxicity, mostly ascribed to electroporation (Fig. 1f and Extended Data Fig. 1c,d). In cord blood (CB)- and mobilized peripheral blood (mPB)-derived CD34^+^ HSPCs, we investigated different timings for gene editing and compared 1 versus 3 d of culture after thawing (Fig. 1g). Whereas a longer protocol promotes metabolic activation and cell cycle progression, a shorter protocol may better preserve stem cell phenotypic markers. *B2M* KO was highly efficient for all systems, with ABE8.20-m outperforming BE4max and Cas9 (reaching up to 88, 63 and 64%, respectively, at the highest dose; Fig. 1h and Extended Data Fig. 1e,f), without detectable changes in the proportion of different progenitor subsets (Fig. 1i and Extended Data Fig. 1g). KO was lower at day 1 than at day 3, in particular for BE4max, Cas9 and the most primitive progenitor subset (Fig. 1h and Extended Data Fig. 1f). HSPCs treated with BE4max and ABE8.20-m showed similar in vitro clonogenic potential as mock-electroporated cells and higher clonogenic potential than Cas9-treated cells, pointing to a milder impact of BEs than Cas9 on HSPC function (Fig. 1j).

We then sequenced the *B2M* target site from the edited cells from Fig. 1h and Extended Data Fig. 1f and found the expected transitions at one or more target bases within the editing window in a proportion of alleles consistent with the fraction of biallelic KO reported above (Fig. 1k and Extended Data Fig. 1h). However, while nearly all ABE8.20-m-edited alleles showed base transitions, more than one-third of BE4max-edited alleles carried indels at the target site. Whereas Cas9-induced indels spanned around the expected DNA DSB site, BE4max indels mostly occurred between the expected nCas9 and BE target sites (Extended Data Fig. 1i). The fraction of indel-bearing alleles was higher for BE4max editing at day 1 than at day 3, when expression of several BER genes, such as *APEX1* and the upstream sensor *UNG*, was also higher (Fig. 1l,m). These findings suggest that excess indels induced by BE4max editing might be due to insufficient UG inhibition by the UGI domain^16^ and the combined action of the BER-dependent APEX1 endonuclease and nCas9 to generate a DSB at the target sequence. Of note, some SNVs other than the expected transitions (‘Other’ in Fig. 1k and Extended Data Fig. 1h) were also found at the target locus for both BEs, as also reported in other studies of CBEs^19,28^, suggesting occasional and/or supplemental engagement of alternative repair pathways.

To provide a broader representation of target sequence composition, including for the number and position of editable bases, additional gRNAs targeting the genomic safe harbor adeno-associated virus site 1 (*AAVS1*), exon 1 of *B2M* and the therapeutically relevant *BCL11A* erythroid-specific enhancer, *CCR5* and *IL2RG* were selected (Fig. 1n). At nearly all tested loci, ABE8.20-m outperformed BE4max in terms of efficiency and precision at the target site (Fig. 1o). While indels and other unexpected SNVs were relatively frequent and more common for BE4max, some indels were also retrieved for ABE8.20-m, in particular when targeting exon 1 of *B2M*. The higher occurrence of indels at the *B2M* exon 1 site allowed to describe the deletions profile of ABE8.20-m and revealed a distribution centered on the gRNA cut site, similar to Cas9, implying a different mechanism from that postulated above for BE4max and likely due to conversion from an SSB to a DSB after DNA replication (Extended Data Fig. 1j). Consistent with the lower proportions of indels, the fraction of primitive HSPCs was not affected by ABE8.20-m treatment, while it was significantly decreased after BE4max and Cas9 treatment (Fig. 1p).

Overall, these data show that highly efficient base editing allows for the emergence of imprecise outcomes at the target sites, comprising the marks of repaired DSBs. These events are exacerbated in the case of BE4max by its interaction with the BER pathway.

### Base editing does not abrogate large deletions and translocations at target sites

To more comprehensively evaluate the spectrum of genetic outcomes at target sites of different editing systems, we screened ∼300 randomly picked colonies from the outgrowth of BE4max-, ABE8.20-m- and Cas9-treated mPB-derived HSPCs for the occurrence of large deletions extending upstream or downstream of *B2M* exon 2 or 1 gRNA target sites (Extended Data Fig. 1h). For *B2M* exon 2 targeting, we found mono- or, less frequently, biallelic loss of the interrogated locus in ∼12% of Cas9 colonies and ∼5% of BE4max colonies but only rarely in ABE8.20-m colonies (Fig. 1q). Of note, a higher proportion of deletions was found when probing downstream of the BE4max cut site, in line with the skewed indels pattern (Extended Data Fig. 1i). For *B2M* exon 1 targeting, we found ∼15% of Cas9 colonies and ∼3% of ABE8.20-m colonies but only rarely in BE4max colonies, where the ABE8.20-m data are consistent with a high proportion of indels at this site, and the BE4max data reflect a low editing efficiency (Fig. 1r and see also Fig. 1o).

We then probed for the possible occurrence of translocations between multiplex editing sites on different chromosomes by co-delivering two gRNAs targeting *AAVS1* and *B2M* exon 2 together with each editing system and amplifying interchromosomal junctions by a matrix of PCR primers binding to each side of both editing loci (Extended Data Fig. 1l). As expected from the high rate of indels and large deletions, Cas9-treated samples were positive for all four possible translocation events between the two sites (Fig. 1s and Extended Data Fig. 1m–o). Notably, translocations were also clearly detectable for BE4max samples in four of six tested donors and for two of the four possible interchromosomal junctions, but not for ABE8.20-m. Sanger sequencing of *B2M*–*AAVS1* junctions revealed that although Cas9 translocations originated precisely from the respective cut sites, BE4max translocations were more heterogenous and spanned from the predicted nCas or APEX1 nicking sites on either side of the junction (Extended Data Fig. 1p).

Overall, these results highlight the occurrence of potentially genotoxic outcomes at BE target sites consequent to DNA DSBs, such as large (greater than or equal to hundreds of base pairs (bp)) deletions and translocations, at rates lower than observed for Cas9 and consistent with the fraction of indels detected by targeted deep sequencing.

### BEs trigger p53 activation and interferon response in HSPCs

**Fig. 2:**
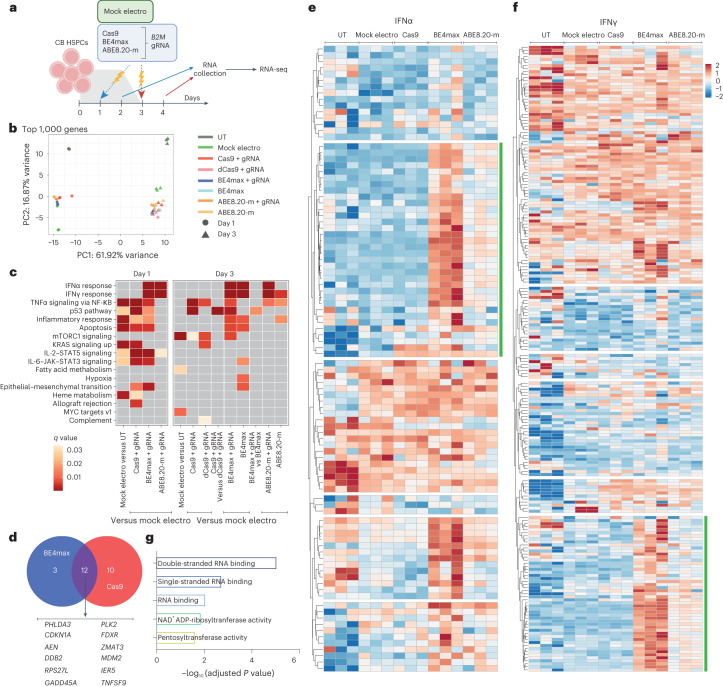
BE mRNAs induce transcriptional responses in human HSPCs. **a**, Experimental workflow for *B2M* exon 2 editing in a pool of six CB HSPC donors for transcriptomic analysis (*n* = 3 technical replicates for each condition). **b**, Principal component (PC) analysis from the RNA-seq dataset in **a**. **c**, Heat map of *q* values of enriched categories for selected comparisons between treatments on upregulated genes (false discovery rate (FDR) < 0.05 and log (fold change) > 0); dCas9, catalytically inactive (dead) Cas9. Data were analyzed by enrichment test. **d**, Venn diagram representing the number of p53 target genes upregulated after BE4max or Cas9 treatment. **e**,**f**, Heat maps of normalized read counts for target genes belonging to IFNα (**e**) and IFNγ (**f**) response categories across samples. Green lines indicate the subset of genes identified by unsupervised clustering with higher normalized read counts after BE treatments. **g**, Adjusted *P* values for the top five enriched categories (Hallmark gene set) when computing genes belonging to the green cluster from **e** and **f**. Data were analyzed by enrichment test. All statistical tests are two tailed. *n* indicates the number of independent samples.

We then investigated the cellular transcriptome 24 h after treatment with the different editors to identify detrimental responses that may impact HSPC function (Fig. 2a). Besides positive enrichment for genes belonging to apoptosis and inflammation categories in all samples due to electroporation per se, BE4max and Cas9 triggered p53 pathway activation (Fig. 2b,c), with upregulation of nearly identical sets of genes, pointing toward sensing and repair of DNA DSBs as the common trigger (Fig. 2d). The p53 response was lower for BE4max than for Cas9, consistent with the above findings for indels and large deletions at the editing site, but still raising concern for a detrimental impact on HSPC function. In addition, BE4max and ABE8.20-m, but not Cas9, activated interferon alpha (IFNα) and IFNγ responses (Fig. 2c). Unbiased clustering of IFNα and IFNγ target genes revealed upregulated subsets after BE treatments enriched for RNA recognition ontologies, possibly indicating innate cellular sensing of long mRNAs (∼6 kilobases; Fig. 2e–g).

### BE4max, but not ABE8.20-m, impairs long-term engraftment of edited HSPCs

**Fig. 3:**
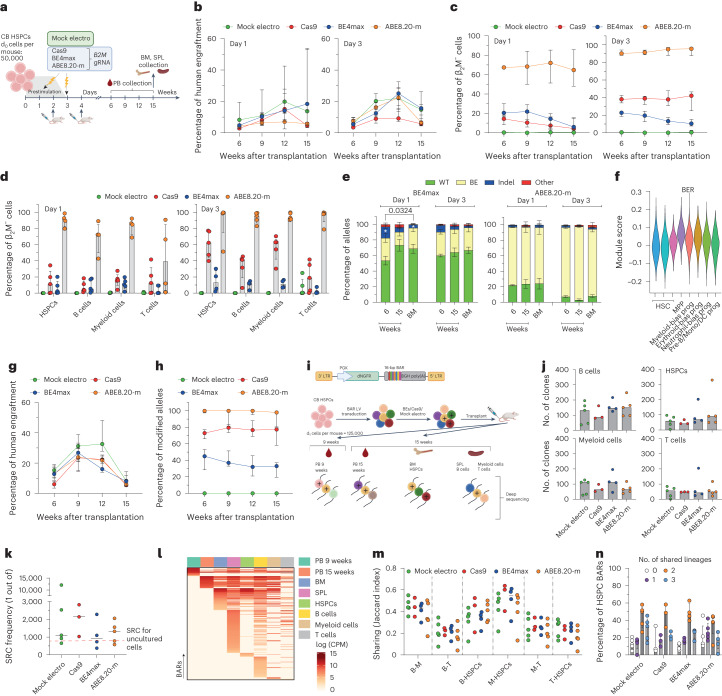
Base editing preserves long-term multilineage repopulation capacity of HSPC clones. **a**, Experimental workflow for *B2M* exon 2 editing in CB HSPCs and xenotransplantation. **b**,**c**, Percentage of human cell engraftment (**b**) and β_2_M^−^ cells within human grafts (**c**) in mice transplanted with CB HSPCs after *B2M* exon 2 editing at day 1 (left; *n* = 5, 5, 5 and 4) or day 3 (right; *n* = 5, 5, 4 and 5) after thawing. Data are shown as median values with IQR and were analyzed by LME model followed by post hoc analysis. **d**, Percentage of β_2_M^−^ cells within hematopoietic lineages from BM (HSPCs) and SPL (B cells, myeloid cells and T cells) from **a** (*n* = 5, 5, 5 and 4 for day 1; *n* = 5, 5, 4 and 5 for day 3). Data are shown as median with IQR. **e**, Percentage of *B2M* exon 2 alleles measured by deep sequencing (WT or carrying the described editing outcomes in mice from **a**; BE4max *n* = 5 for day 1, *n* = 4 for day 3; ABE8.20-m *n* = 4 day 1, *n* = 5 for day 3). Data are shown as mean ± s.e.m. and were analyzed by Kruskal–Wallis with Dunn’s multiple comparison. **f**, Module score for genes belonging to the BER pathway (KEGG database hsa03410) in different HSPC subsets from Schiroli et al.^5^; MPP, multipotent progenitors; prog, progenitors; Mono, monocyte; DC, dendritic cell; Pre-B, pre-B cell. **g**,**h**, Percentage of human cell engraftment (**g**) and modified *AAVS1* alleles within human grafts (**h**) in mice transplanted with mPB HSPCs after *AAVS1* editing at day 3 after thawing (*n* = 4, 4, 5 and 5). Data are shown as median with IQR and were analyzed by LME model followed by post hoc analysis. **i**, Schematic representation of the barcoded LV library (top) and the workflow for the CB HSPC clonal tracking experiment (bottom); LTR, long-terminal repeat; PGK, phosphoglycerate kinase promoter; BGH, bovine growth hormone polyadenylation signal. ‘+’ is used to graphically mark edited cells. **j**, Number of clones in hematopoietic lineages from mouse organs from **i** (*n* = 5, 3, 4 and 5). Data are shown as median values. **k**, Severe combined immunodeficient (SCID)-repopulating cell (SRC) frequency in mice from **i**, calculated by dividing the d_0_ equivalent cell number by the number of engrafted clones from BM in Extended Data Fig. 2p. The red line shows the SRCs for uncultured HSPCs^29^ (*n* = 5, 3, 4 and 5). Data are shown as median values. **l**, Heat map of the abundance (red-scaled palette) of BARs (rows) for one representative BE4max mouse in PB at the indicated times after transplant, hematopoietic organs and lineages (columns). **m**, Jaccard index as a measure of BAR sharing between B cells and myeloid cells (B-M); B and T cells (B-T); B cells and HSPCs (B-HSPCs); myeloid cells and HSPCs (M-HSPCs); myeloid and T cells (M-T); T cells and HSPCs (T-HSPCs) (*n* = 5, 3, 4 and 5). Data are shown as median values. **n**, Percentage of unique HSPC BARs shared with 0, 1, 2 or 3 hematopoietic lineages (*n* = 5, 3, 4 and 5). Data are shown as median values with IQR. All statistical tests are two tailed. *n* indicates independent animals.

**Extended Data Fig. 2:**
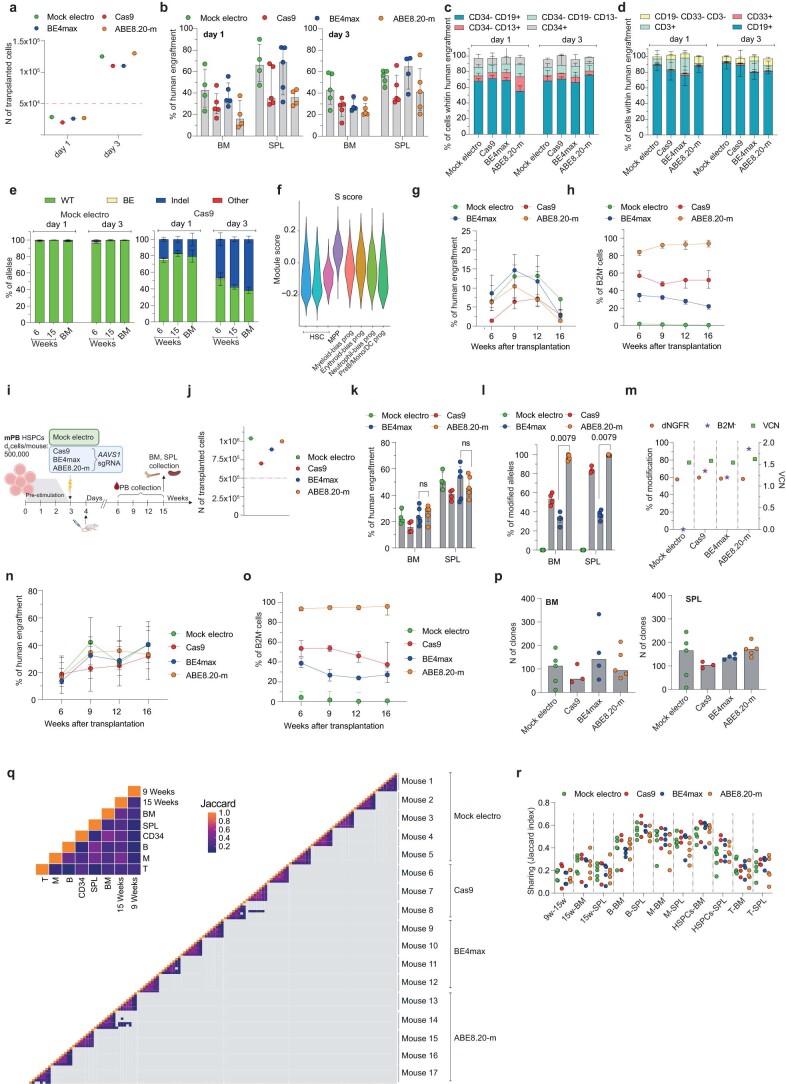
In vivo persistence of edited HSPCs. **a**, Number of cells transplanted per mouse for experiment in Fig. 3a. The red line corresponds to the d_0_ equivalent. **b-d**, Percentage of human cells engraftment in BM and SPL (b; median with IQR) and their respective lineage composition (c,d; mean $\pm$ s.e.m) in mice from Fig. 3a (n = 5,5,5,4 for day 1; n = 5,5,4,5 for day 3). **e**, Percentage of *B2M* exon 2 alleles, measured by deep sequencing, being WT or carrying the described editing outcomes in mice from Fig. 3a (Mock electro n = 4 for day 1, n = 5 for day 3; Cas9 n = 5 day 1, n = 5 for day 3). Mean $\pm$ s.e.m. **f**, Module score for genes belonging to the S-phase signatures from^64^ in different HSPC subsets from^5^. **g,h**, Percentage of human cells engraftment (g) and B2M^−^ cells within human graft (h) in mice transplanted with mPB HSPCs edited at *B2M* exon 2 at day 3 post-thawing (n = 4,5,6,5). Median with IQR. LME followed by post hoc analysis. **i**, Experimental workflow for *AAVS1* editing in mPB HSPCs and xenotransplantation. **j**, Number of cells transplanted per mouse for experiment in ‘i’. The red line corresponds to the d_0_ equivalent. **k,l**, Percentage of human cells engraftment (k) and *AAVS1* modified alleles (l) in BM and SPL from ‘i’ (n = 4,4,5,5). Median with IQR. Mann-Whitney test. **m**, Percentage of dNGFR^+^ and B2M^−^ cells, measured by flow cytometry, and vector copy number (VCN), measured by ddPCR. **n,o**, Percentage of human cells engraftment (n) and B2M^−^ cells within human graft (o) in mice from Fig. 3i (n = 5,3,4,5). Median with IQR. **p**, Number of clones in hematopoietic organs of mice from Fig. 3i (n = 5,3,4,5). Median. **q**, Heatmap showing the Jaccard index as a measure of inter- and intra-sample BAR sharing in mice from Fig. 3i. **r**, Jaccard index as a measure of BAR sharing between hematopoietic organs in mice from Fig. 3i (n = 5,3,4,5). Median. All statistical tests are two-tailed. n indicate independent animals.

To investigate editing of the small fraction of repopulating HSPCs comprised within CD34^+^ cells, we transplanted immunodeficient mice with the outgrowth of matched numbers of cells seeded in culture (day 0) and treated for *B2M* exon 2 editing by the different systems at day 1 or 3 (Fig. 3a and Extended Data Fig. 2a). Longitudinal PB sampling and bone marrow (BM) and spleen (SPL) analyses at the end of the experiments showed long-term engraftment and multilineage reconstitution by human cells in xenotransplanted mice for all edited samples (Fig. 3b and Extended Data Fig. 2b–d). Engraftment at 9–12 weeks was higher for day 3- than for day 1-treated cells, likely due to an expanded number of short-term repopulating cells in the longer culture. Across multiple experiments, the graft size was significantly lower (*P* = 0.0075) for Cas9-edited samples than for mock-electroporated samples throughout the follow-up, likely due to the higher DNA DSB load and p53 activation induced by the nuclease^5^, while the base-edited samples were significantly lower than mock-electroporated samples only at the end of the study (*P* = 0.049 and *P* = 0.007, respectively; Supplementary Fig. 1a). When monitoring the edited cell fraction by the proportion of *B2M*-KO cells in the graft, ABE8.20-m maintained the very high levels of editing achieved in culture in vivo, which were stable in the long-term graft, similar to Cas9-edited cells, which, however, remained at lower levels (Fig. 3c,d and Supplementary Fig. 1b). By contrast, BE4max-edited cells showed a much lower level of editing than the in vitro results and further decreased over time in the graft, in line with a detrimental impact of the editor and/or a lower editing efficiency in long-term repopulating HSPCs (Fig. 3c,d and Supplementary Fig. 1b; interaction term at time 15 weeks, *P* < 0.0001). Deep-sequencing analysis of the *B2M* exon 2 target site in human cells retrieved from the mice showed nearly exhaustive occurrence of the expected transitions for the ABE8.20-m samples and a lower proportion consistent with the fraction of engrafted *B2M*-KO cells for the BE4max samples (Fig. 3e). Indels accounted for most of the editing in Cas9-engrafted cells (Extended Data Fig. 2e) but also contributed considerably in the BE4max samples, where they were more abundant in cells edited at day 1 and decreased from early to late timepoints (Fig. 3e). The latter observation might correlate with higher BER gene expression in day 1-cultured cells and in multipotent or lineage-committed progenitors than in primitive HSCs, as reported by some of us in a single-cell RNA-sequencing (RNA-seq) analysis of CD34^+^CD133^+^ CB cells cultured for 4 d (ref. ^5^; Fig. 3f and Extended Data Fig. 2f). The frequency of indels was lower, but indels were still present, in ABE8.20-m samples, averaging 1–2%. Similar findings in terms of engraftment and editing efficiency were obtained when transplanting HSPCs derived from mPB and edited with all systems either at *B2M* exon 2 (Extended Data Fig. 2g,h) or *AAVS1* (Fig. 3g,h and Extended Data Fig. 2i–l).

To investigate more stringently whether base editing could affect the output of single HSPC clones, we tracked cells treated with BEs or Cas9 at the clonal level. Because it is hardly possible to couple BEs to a unique genetic identifier, we transduced HSPCs with a lentiviral vector (LV) carrying a reporter (truncated low-affinity nerve growth factor receptor; dNGFR) and a degenerated barcode sequence (BAR) before editing and xenotransplantation (Fig. 3i and Extended Data Fig. 2m). While this strategy cannot discriminate between edited and non-edited cells in the graft, we reached high proportions of *B2M*-KO cells in vivo for the ABE8.20-m samples, which were virtually all edited (Extended Data Fig. 2n,o). Thus, an altered behavior of ABE8.20-m-edited cells should be easily captured by interrogating the whole graft, while this may not apply to BE4max-treated cells. Although Cas9 treatment led to a moderate shrinkage of clonal complexity in the hematopoietic organs and in most of the sorted hematopoietic lineages, consistent with the lower graft size, ABE8.20-m-edited cells (and BE4max-edited cells) did not show reduced clonality compared to mock-electroporated control cells (Fig. 3j and Extended Data Fig. 2p). Because 100–150 repopulating clones were retrieved from base-edited and mock-treated samples, we calculated a frequency of 1 of 1.0 × 10^3^–1.5 × 10^3^ transplanted cells (Fig. 3k), which is in the range of previously reported findings from limiting dilution transplant of uncultured CB-derived HSPCs^29^. Longitudinal analysis of PB revealed the progressive disappearance of some short-term engrafting clones and the emergence of long-term engrafting clones independent of the treatment, in line with previous observations on HDR-edited xenografts^6^ and individuals treated with gene therapy^30^ (Fig. 3l and Supplementary Fig. 2). Most BARs were shared across the different lineages of the same mouse (Fig. 3m,n and Extended Data Fig. 2q,r).

Overall, these findings show that ABE8.20-m efficiently edits and preserves multilineage output of long-term repopulating HSPCs, while BE4max is less efficient and adversely impacts repopulation by edited cells.

### Transcriptome and exome analyses uncover global effects of BEs on the mutational landscape

**Fig. 4:**
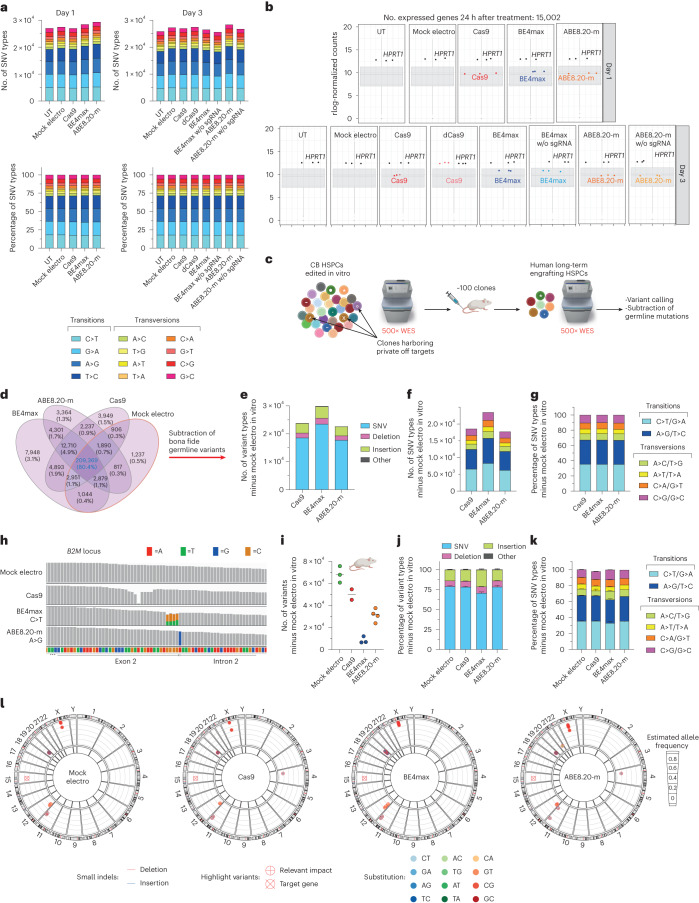
Effects of base editing on the mutational landscape in HSPCs across the transcriptome and genome. **a**, Number of SNV types (top) and their relative proportions (bottom) in RNA-seq experiments in Fig. 2a; w/o, without; sgRNA, single gRNA. **b**, Box plot showing the normalized expression (read counts) of the different editors and the *HPRT1* housekeeping gene in RNA-seq experiments in Fig. 2a; rlog, regularized log. **c**, Schematic representation of the WES rationale and bioinformatic pipeline in CB HSPCs treated in vitro and retrieved from xenotransplanted mice in Fig. 3i. **d**, Venn diagrams representing variants shared among in vitro treated samples from **e**. **e**–**g**, Number of variants (**e**), number of SNV types (**f**) and their relative proportion (**g**) from in vitro samples from **c** obtained after subtraction of germline variants. **h**, Read alignments at *B2M* in the WES dataset from **c**. **i**–**k**, Number of variants (median; **i**), relative proportion of variants (mean ± s.e.m; **j**) and relative proportion of SNV types (mean ± s.e.m.; **k**) in the human xenograft from **c** obtained after subtraction of germline variants (*n* = 3, 2, 3 and 4). **l**, Circos plots representing variants in cancer-associated genes classified as high/moderate impact identified by WES in the human xenografts from **c** (*n* = 3, 2, 3 and 4). All statistical tests are two tailed. *n* indicates the number of independent samples for Fig. 1a,b and independent animals for **i**–**l**.

Next, we evaluated the mutational burden induced by BEs at both transcriptomic and genomic levels. We found a consistent, albeit moderate, increase in mutational load on the transcriptomes of HSPCs edited at day 1 or day 3 by ABE8.20-m compared to all other treatments despite similar levels of editor expression (Fig. 4a). The increase applied to all SNV types and not only to the expected A>G transition (Fig. 4b).

We then explored the possible occurrence of gRNA-independent genome-wide DNA mutagenesis by the constitutively active, but transiently expressed, deamination domain of the tested BEs on chromatin R-loops. We performed ultrahigh coverage (500×) whole-exome sequencing (WES) of the in vitro outgrowth of HSPCs treated with the different editors from the experiment described in Fig. 3i, calling all variants against the reference human genome (GRCh38) and plotting their intersections among all samples (Fig. 4c,d). As expected, the vast majority of variants were shared by all samples, reflecting germline variants of the HSPC donors. We then subtracted all variants shared between mock-electroporated samples and ≥1 sample from each condition to capture treatment-associated variants and found that BE4max treatment increased their total amounts, but not the relative proportions of different SNVs, compared to Cas9 or ABE8.20-m treatment (Fig. 4e–g). We then postulated that analyzing the expanded clonal outgrowths contributing to the oligoclonal human hematopoietic graft of transplanted mice might increase the sensitivity of analysis toward detection of non-recurring genome-wide variants acquired by individual long-term repopulating cells during the treatment, albeit at the cost of limited sampling (Fig. 4c). The expected C>T or A>G transitions were highly represented at the *B2M* target locus in all BE4max and ABE8.20-m samples, respectively, thus validating our pipeline (Fig. 4h). Similarly, for Cas9 samples, we retrieved substantial proportions of indels in the target region, reflected by drops in read alignment. For the genome-wide analysis, as before, we subtracted all variants previously called for the mock-electroporated samples in the in vitro analysis (Fig. 4d) and computed those specific for each mouse/HSPC treatment. Remarkably, we found the lowest figures for BE4max samples, followed by ABE8.20-m, Cas9 and mock-electroporated samples in order of increasing numbers (Fig. 4i). This pattern was reminiscent of the impact of treatment on edited cell engraftment, as shown in Fig. 3c,h and Extended Data Fig. 2h,o, with variant diversity being a proxy for clonality. However, when we computed the different types of variants and relative proportions of SNV types, we found a similar pattern among ABE8.20-m, Cas9 and mock samples and a slight increase of indels and lower proportions of C>T/G>A transitions and higher proportions of A>C/T>G and G>C/C>G transversions in the BE4max samples, implying a treatment-specific effect on the mutational landscape (Fig. 4j,k). When annotating high- and moderate-impact variants within a selected panel of cancer-associated genes, which may provide a selective advantage to mutant clones, we found few variants in all treated samples, most of which were shared among all treatment groups (Fig. 4l and Supplementary Table 1).

In summary, at the genome-wide level, treatment with BE4max showed alteration of the exome mutational landscape compared to mock or other editing treatments, with an increased load in bulk-analyzed in vitro outgrowth of treated HSPCs and a substantial drop in the oligoclonal resulting graft. Notably, the latter observation was accompanied by a skewed distribution disfavoring the expected deaminase-induced transitions.

### Optimized mRNA design improves efficiency and precision of base editing at target sites

**Fig. 5:**
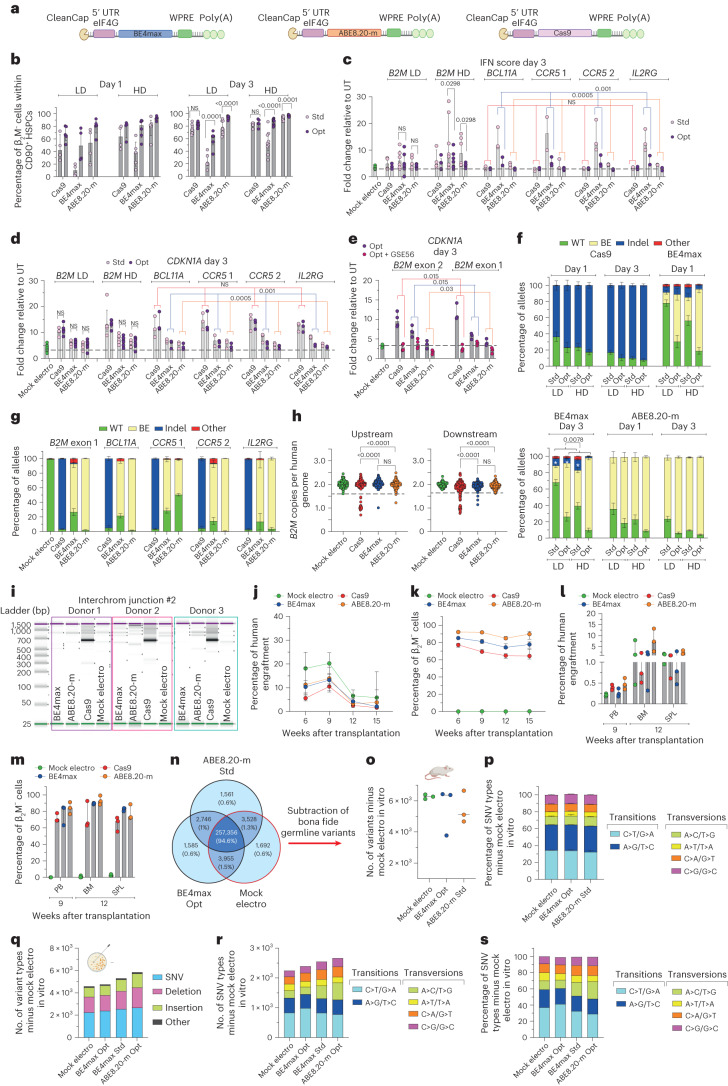
Optimized BE mRNAs improved editing efficiency and precision at the target site, dampened cellular responses but perturbed the mutational landscape after increased expression. **a**, Schematic representation of the optimized mRNAs. **b**, Percentage of β_2_M^−^CD34^+^CD133^+^CD90^+^ mPB HSPCs after editing at day 1 (left) or day 3 (right) after thawing and measured by flow cytometry; Std/Opt, standard/optimized; LD, low dose (3.5 μg); HD, high dose (7.5 μg; *n* = 4, 5, 4, 4, 4 and 5 for low dose day 1; *n* = 4, 4, 5, 6, 5 and 5 for high dose day 1; *n* = 8, 9, 6, 6, 8 and 9 for low dose day 3; *n* = 5, 5, 9, 10, 7 and 7 for high dose day 3). Data are shown as median with IQR and were analyzed by LME model followed by post hoc analysis. **c**, IFN score defined as the sum of fold change of *IRF7*, *OAS1* and *DDX58* expression over untreated samples 24 h after editing at day 3 after thawing (*n* = 9 for mock-electroporated samples; *n* = 4, 5, 6, 5, 5 and 5 for *B2M* low dose; *n* = 4, 5, 7, 7, 6 and 5 for *B2M* high dose; *n* = 3 for *BCL11a*, *CCR5* and *IL2RG*). Data are shown as median values with IQR. For *B2M* low dose and high dose, data were analyzed by LME followed by post hoc analysis. For *BCL11a*, *CCR5* and *IL2RG*, data were analyzed by Friedman test with a Dunn’s multiple comparison on unified samples. **d**, Fold change of *CDKN1A* expression over untreated samples 24 h after editing at day 3 after thawing (*n* = 11 for mock-electroporated samples; *n* = 5, 5, 6, 6, 6 and 6 for *B2M* low dose day 3; *n* = 5, 5, 7, 7, 7 and 7 for *B2M* high dose day 3; *n* = 3 for *BCL11a*, *CCR5* and *IL2RG*). Data are shown as median values with IQR. For *B2M* low dose and high dose, data were analyzed by LME model followed by post hoc analysis. For *BCL11A*, *CCR5* and *IL2RG*, data were analyzed by Friedman test with Dunn’s multiple comparison test on unified samples. **e**, Fold change of *CDKN1A* expression over untreated samples 24 h after editing at day 3 after thawing with optimized mRNA in the absence or presence of GSE56 (*n* = 3). Data are shown as median with IQR and were analyzed by Wilcoxon test on the *B2M* exon 1 and exon 2 unified samples. **f**, Percentage of *B2M* exon 2 edited alleles measured by deep sequencing (WT or carrying the described editing outcomes; *n* = 4). Data are shown as mean ± s.e.m. and were analyzed by Wilcoxon test performed on day 3 ‘Std’ versus ‘Opt’ groups unifying mRNA doses for statistical analysis. **g**, Percentage of *B2M* exon 1 (*n* = 4), *BCL11A*, *CCR5* and *IL2RG* (*n* = 3) edited alleles measured by deep sequencing (WT or carrying the described editing outcomes). Data are shown as mean ± s.e.m. **h**, Copies of *B2M* sequences per human genome flanking the target site in individual colonies generated by edited mPB HSPCs using optimized mRNAs (*n* = 105, 186, 184 and 185 for the ‘upstream’ assay; *n* = 93, 188, 187 and 186 for the ‘downstream’ assay). Dashed lines indicate the lower limit of the confidence interval from mock-electroporated colonies. Data are shown as median with IQR and were analyzed by Fisher’s exact test. **i**, Images of capillary electropherogram showing amplification of interchromosomal junction 2 shown in Extended Data Fig. 1l after HSPC editing with two gRNAs targeting *B2M* exon 2 and *AAVS1* in three mPB donors. **j**,**k**, Percentage of human cell engraftment (**j**) and percentage of β_2_M^−^ cells within human grafts (**k**) in mice transplanted with mPB HSPCs edited at day 3 after thawing with optimized Cas9, BE4max and ABE8.20-m mRNAs at the lowest maximally effective dose (3.5, 7.5 and 3.5 µg, respectively; *n* = 6). Data are shown as median values with IQR and were analyzed by LME model followed by post hoc analysis. **l**,**m**, Percentage of human cell engraftment (**l**) and β_2_M^−^ cells within human grafts (**m**) in secondary recipient mice from **j** (*n* = 3). Data are shown as median with range. **n**, Venn diagrams representing variants shared among in vitro treated samples from Extended Data Fig. 3k. **o**,**p**, Number of variants (median; **o**) and relative proportion of SNV types (mean ± s.e.m; **p**) in the human xenograft from Extended Data Fig. 3k obtained after subtraction of germline variants. **q**–**s**, Number of variants (**q**), number of SNV types (**r**) and their relative proportion (**s**) in the pool of colonies from Extended Data Fig. 3m obtained after subtraction of germline variants. All statistical tests are two tailed. *n* indicates biologically independent experiments except for **h** and **q**–**s**, in which *n* indicates independent samples, and **j**–**p**, in which *n* indicates independent animals.

**Extended Data Fig. 3:**
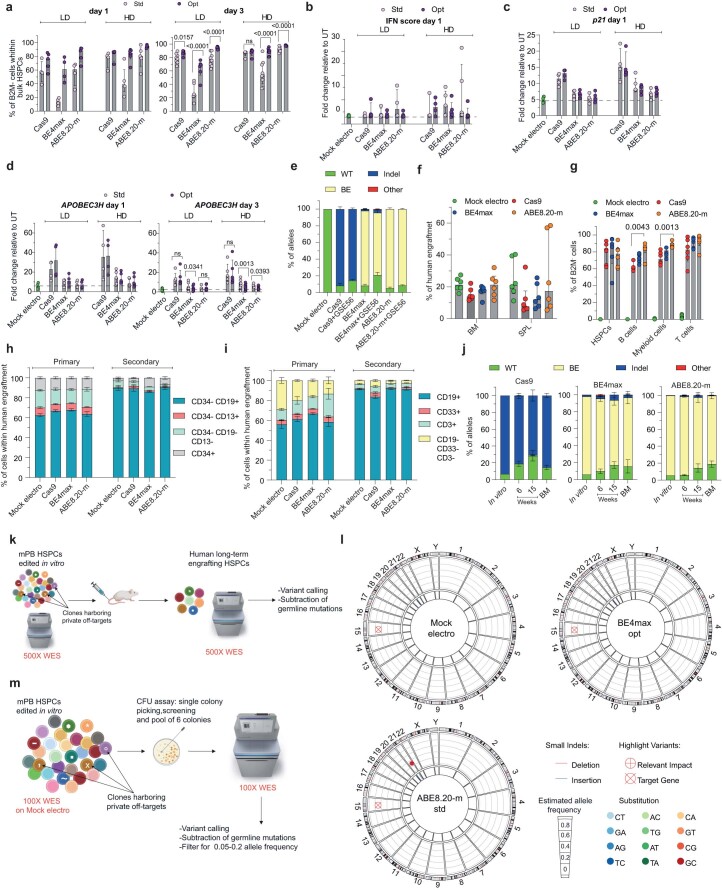
Impact of editors’ mRNA optimization on editing efficiency, cellular responses and precision at the target site and genome wide. a, Percentage of B2M^−^ HSPCs after editing at day 1 (left) or day 3 (right) post-thawing, measured by flow cytometry, from Fig. 5b (n = 4,5,4,4, 4,5 for LD day 1; n = 4,4,5,6,5,5 for HD day1; n = 8,9,6,6,8,9 for LD day 3; n = 5,5,9,10,7,7 for HD day 3). Median with IQR. LME followed by post hoc analysis. b, IFN score defined as sum of fold change of IRF7, OAS1 and DDX58 expression over UT 24 hrs after editing at day 1 post-thawing (n = 3 for Mock electro; n = 3,4,4,4,4,4 for LD; n = 3,4,5,5,5,5 for HD. Median with IQR. c, Fold change of p21 expression over UT 24 h after editing at day 1 post-thawing (n = 4 for Mock electro; n = 4 for LD; n = 4,4,5,5,4,5 for HD). Median with IQR. d, Fold change of APOBEC3H expression over UT 24 h after editing at day1 (left) and day 3 (right) post-thawing (n = 4 for Mock electro day 1; n = 4 for LD day 1; n = 4,4,5,5,5,5 for HD day 1; n = 8 for Mock electro day 3; n = 5,5,6,6,6,6 for LD day 3; n = 5,5,7,7,7,7 for HD day 3). Median with IQR. LME followed by post hoc analysis. e, Percentage of B2M exon 2 edited alleles, measured by deep sequencing, being WT or carrying the described editing outcomes (n = 3). Mean ± s.e.m. f,g, Percentage of human cells engraftment (f) and B2M^−^ cells within hematopoietic lineages (g) in BM and SPL in mice form Fig. 5j (n = 6). Median with IQR. Kruskal-Wallis with Dunn’s multiple comparison. h,i, Lineage composition in BM (h) and SPL (i) in mice from Fig. 5j and Fig. 5l (n = 6,6,6,6,3,3,3,3). Mean ± s.e.m. j, Percentage of B2M exon 2 alleles, measured by deep sequencing, being WT or carrying the described editing outcomes in mice from Fig. 5j and in the in vitro outgrowth (In vitro: n = 1; Cas9: n = 5; BE4max: n = 6; ABE8.20-m: n = 6). Mean ± s.e.m. k, Schematic representation of the WES rationale and bioinformatic pipeline in mPB HSPCs treated in vitro and retrieved from xenotransplanted mice. l, Circos plots representing variants in cancer associated genes classified as high/moderate impact identified by WES by WES in the human xenograft from k. m, Schematic representation of the WES rationale and bioinformatic pipeline in mPB HSPCs treated in vitro and retrieved from pool of individual colonies. All statistical tests are two-tailed. n indicate biologically independent experiments except for Extended Data Fig. 3e–l in which n indicate independent animals and Extended Data Fig. 3m in which n indicate independent samples.

To investigate whether the worse performance and lower precision of BE4max could be improved by enhancing expression and lowering innate sensing, we engineered the mRNA constructs with a 5′ cap, better recapitulating the endogenous structure, and included a eukaryotic initiation factor 4G (eIF4G) aptamer in the 5′-untranslated region (5′-UTR; Fig. 5a). Using these optimized mRNAs, we could decrease the effective mRNA dose and reach equivalent or superior editing efficiencies for all editing systems in both bulk and primitive HSPCs (Fig. 5b and Extended Data Fig. 3a), nearly abolishing activation of the IFN response (Fig. 5c and Extended Data Fig. 3b) and lowering the p53 response across different target loci (Fig. 5d and Extended Data Fig. 3c,d). When the optimized editor mRNAs targeting *B2M* were co-delivered with an mRNA encoding the p53 dominant-negative mutant GSE56, we abrogated p21 (*CDKN1A*) induction for all editors tested (Fig. 5e), albeit at the cost of slightly reduced efficiency and increased proportion of indels at the target site for BE4max (Extended Data Fig. 3e). By contrast, indels induced at the target site by BE4max were significantly reduced when comparing optimized mRNAs to standard mRNAs (Fig. 5f,g compared to Fig. 1o because experiments were performed side by side with cells from the same HSPC donors). The apparent paradoxical decrease in indels with increased intended base editing by BE4max might be explained by higher coexpression of the UGI domains, resulting in stronger inhibition of BER-initiating factors. Consistent with this hypothesis, the proportion of indels was further lowered when editing at day 3 versus at day 1, when BER-associated genes are less expressed and when using higher doses of mRNA. We obtained similar findings by screening 200 randomly picked HSPC-derived colonies for the occurrence of large deletions encompassing the *B2M* exon 2 target site (Fig. 5h). There were fewer colonies bearing large deletions from cells edited at day 3 with optimized BE4max mRNA than with standard BE4max mRNA, while Cas9-treated colonies showed, as expected, the opposite behavior (Fig. 5h compared to Fig. 1q because experiments were performed side by side with cells from the same HSPC donors). Similarly, translocations were not detected when using optimized BE4max mRNA, whereas they were again found in Cas9-treated samples (Fig. 5i compared with Fig. 1s because experiments were performed side by side with cells from the same HSPC donors).

Using the optimized mRNAs at the lowest maximally effective dose allowed reaching >90% stable frequency of edited cells in the mouse xenografts for ABE8.20-m treatment and nearly 80% for BE4max treatment (Fig. 5j,k and Extended Data Fig. 3f–i). These levels of edited cells were also maintained in the human graft of secondary transplants (Fig. 5l,m and Extended Data Fig. 3h,i). Indels at the edited site were low, albeit still detectable, in the human graft of primary recipients for both BEs, confirming that the optimized mRNAs increased not only efficiency but also precision of genetic outcome in long-term repopulating HSPCs (Extended Data Fig. 3j).

### Perturbation of the exome mutational landscape emerges after increased expression of BEs

We then evaluated whether the improved expression and activity of BE4max impacted the genome-wide mutational landscape of treated cells and performed the same analyses described above in Fig. 4c (Extended Data Fig. 3k). Different from before, the total number of treatment-associated sequence variants was similar for optimized BE4max, standard ABE8.20-m and mock-electroporated samples (Fig. 5n). Moreover, when we analyzed long-term engrafting clones, subtracting the donors’ germline variants identified in the in vitro analysis, we found similar median numbers of variants among BE4max optimized mRNA-treated and mock-electroporated mice and a slight reduction in ABE8.20-m standard mRNA-treated mice. No differences were observed in the relative proportions of SNV types among all transplanted mice and in the number of variants retrieved in cancer-associated genes (Fig. 5o,p and Extended Data Fig. 3l).

These findings confirm a specific vulnerability of the BE4max editor, likely due to insufficient inhibition of the BER pathway, which results in loss of edited cells and oligoclonal grafts and is alleviated by improved expression of the editor. However, a concern remains that the impact of BE4max on the genomic mutational landscape that emerged in the prior condition might now have escaped detection because of limited sensitivity in the context of more robust clonal abundance in the sample. We thus performed an orthogonal analysis on samples comprising a small, known and evenly distributed number of edited clones (Extended Data Fig. 3m). We sequenced the exomes of pools of six edit-bearing colonies outgrown from single-donor HSPCs treated with each different editor and expression construct and focused our bioinformatic analysis on mutations with a variant allele frequency compatible with the rate of in vitro accrual of mutations. Remarkably, this analysis confirmed previous findings of a slight increase in, but evident skewing of, SNV types toward transversions for standard BE4max samples compared to mock samples, which was alleviated by the improved expression construct (Fig. 5q–s). Notably, in the latter optimized conditions, a trend toward increased proportions of the expected C>T/G>A transitions emerged over mock-electroporated samples. However, cells treated with optimized ABE8.20-m construct showed an even higher increase in variants, with skewed proportions toward transversions, cautioning that increased and/or prolonged activity of this type of editor might also increase adverse effects across the genome.

### Efficient prime editing in human HSPCs does not escape DNA DSBs and cellular sensing

**Fig. 6:**
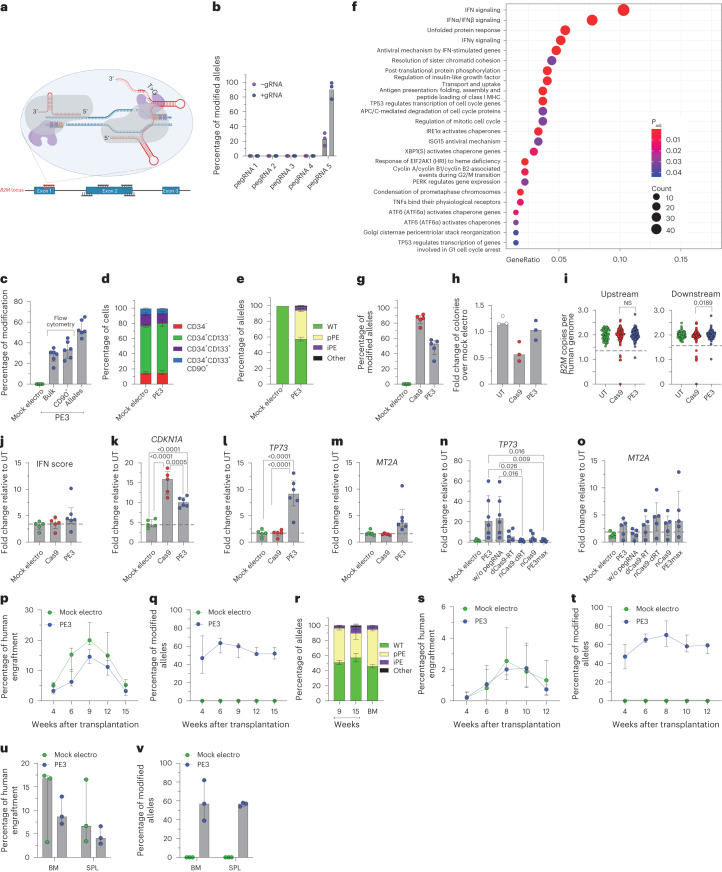
Efficient prime editing in long-term repopulating HSPCs. **a**, Schematic representation of the *B2M* prime editing screening. The selected pegRNA and gRNA are represented in red. **b**, Percentage of *B2M* prime-edited alleles in K-562 cells measured by Sanger sequencing 9 d after the editing procedure (*n* = 3). Data are shown as median values. **c**, Flow cytometry (bulk and CD90^+^) and molecular analysis of *B2M* modification 7 d after prime editing in human mPB HSPCs (*n* = 6). Data are shown as median values with IQR. **d**, Proportion of cell subpopulations within mPB HSPCs from experiments in **c** (*n* = 6). Data are shown as mean ± s.e.m. **e**, Percentage of *B2M* alleles measured by deep sequencing (WT or carrying precise prime editing (pPE), imprecise prime editing (iPE) or other modifications in mPB HSPCs; *n* = 6). Data are shown as mean ± s.e.m. **f**, Dot plot of adjusted *P* values of enriched categories on upregulated (FDR < 0.05 and log (fold change) > 0) genes for PE3 versus mock-electroporated HSPCs. Data were analyzed by enrichment test; *P*_adj_, adjusted *P* value; MHC, major histocompatibility complex. **g**, Percentage of *B2M* Cas9- or PE3-edited alleles 7 d after treatment of mPB HSPCs (*n* = 5). Data are shown as median values with IQR and were analyzed by Mann–Whitney test. **h**, Fold change in the number of colonies generated by mPB HPSCs over mock-electroporated cells (*n* = 3). Data are shown as median values. **i**, Copies of *B2M* sequences per human genome flanking the target site in individual colonies generated by edited mPB HSPCs (*n* = 70, 137 and 137 for the ‘upstream’ assay; *n* = 70, 137 and 139 for the ‘downstream’ assay). Dashed lines indicate the lower limit of the confidence interval from mock-electroporated colonies. Data are shown as median values with IQR and were analyzed by Fisher’s exact test. **j**, IFN score defined as the sum of fold change of *IRF7*, *OAS1* and *DDX58* expression over untreated 24 h after editing (*n* = 5, 5 and 6). Data are shown as median with IQR and were analyzed by LME followed by post hoc analysis. **k**–**o**, Fold change in expression of *CDKN1A* (*n* = 5, 5 and 6; **k**), *TP73* (*n* = 5, 5 and 6; *n* = 5, 6, 6, 5, 5, 5 and 5; **l**) and *MT2A* (*n* = 5, 5 and 6; *n* = 5, 6, 6, 5, 5, 5 and 5; **m**) over that in untreated samples 24 h after editing (*n* = 5, 5 and 6). Data are shown as median values with IQR and were analyzed by LME model followed by post hoc analysis; dRT, catalytically inactive (dead) RT. **p**,**q**, Percentage of human cell engraftment (LME model followed by post hoc analysis; **p**) and percentage of modified *B2M* alleles within human grafts (**o**) in mice transplanted with mPB HSPCs edited at *B2M* following PE3 or mock electroporation (*n* = 5 and 6). Data are shown as median with IQR. **r**, Percentage of *B2M* alleles measured by deep sequencing (WT or carrying precise prime editing, imprecise prime editing or other modifications in PB and BM of mice from **p**; *n* = 6). Data are shown as mean ± s.e.m. **s**,**t**, Percentage of human cell engraftment (**s**) and percentage of modified *B2M* alleles within human grafts (**t**) in secondary recipient mice (*n* = 3). Data are shown as median values with ranges. **u**,**v**, Percentage of human cell engraftment (**u**) and modified *B2M* alleles (**v**) in BM and SPL of secondary recipient mice from **s** (*n* = 3). Data are shown as median values with ranges. All statistical tests are two tailed. *n* indicates biologically independent experiments except for **i**, in which *n* indicates independent samples, and **p**–**v**, in which *n* indicates independent animals.

**Extended Data Fig. 4:**
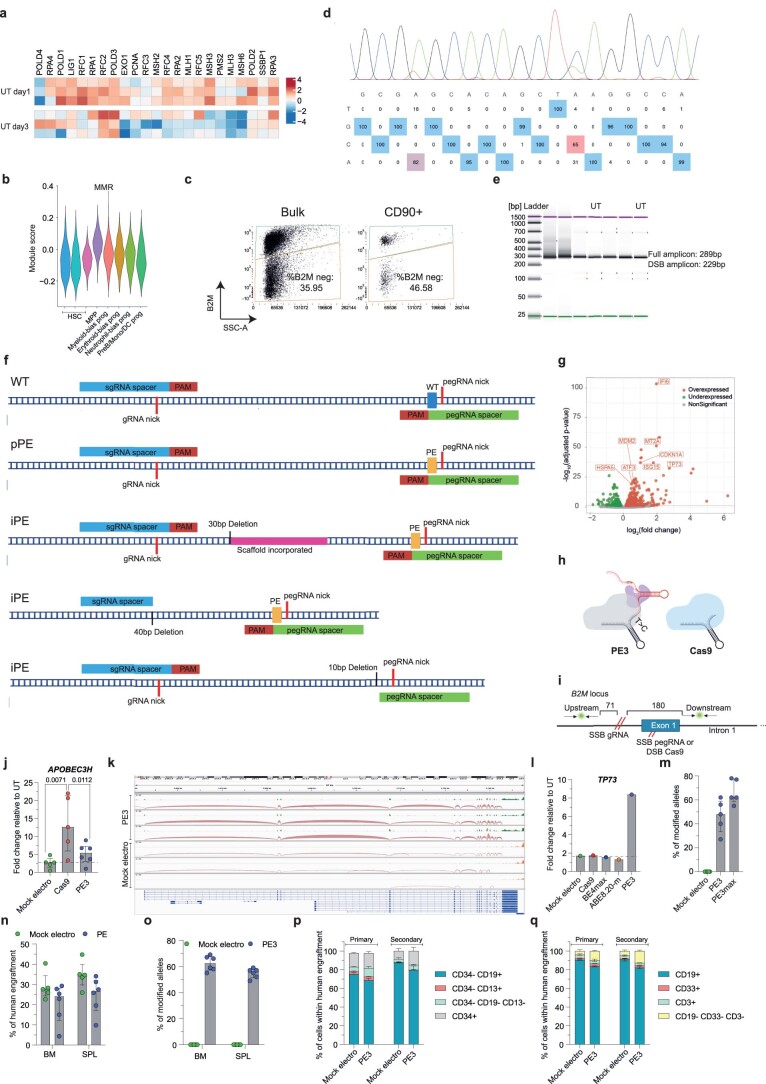
Prime editing in human HSPCs and its adverse impact on HSPCs. **a**, Heatmap of normalized read counts for genes belonging to the MMR pathway (KEGG database; hsa03430) in UT CB HSPCs cultured for 1 or 3 days. **b**, Module score for genes belonging to the MMR pathway in different HSPC subsets from^5^. **c**, Flow cytometry plots showing the gating strategy for prime edited B2M^−^ mPB HSPCs. **d**, Representative plot of *B2M* Sanger sequencing. **e**, Representative image of capillary electropherogram of prime edited (n = 5) and UT (n = 2) samples. **f**, Schematics of representative alleles for WT, pPE and iPE outcomes in prime edited samples from Fig. 6e. **g**, Volcano plot showing significant down- (green) and up- (red) regulated genes after prime editing. **h**, Schematic representation of the spacer sequence shared between pegRNA and gRNA for Cas9. **i**, Schematic representation of the probes used for deletions detection. The distances between the target site and the closest primer of the ddPCR amplicons are shown. **j**, Fold change of *APOBEC3H* expression over UT 24 hrs after editing (n = 5,5,6). Median with IQR. LME followed by post hoc analysis. **k**, Representation of *TP73* spliced alignment from RNA-Seq reads. **l**, Fold change of *TP73* expression over UT 24 hrs after editing (n = 1). **m**, Molecular analysis of *B2M* modification 7 days after prime editing in human mPB HSPCs (n = 5). Median with IQR. **n,o**, Percentage of human cells engraftment (n) and *B2M* modified alleles (o) in BM and SPL of mice from Fig. 6p (n = 5,6). Median with IQR. **p,q**, Lineage composition in BM (p) and SPL (q) of mice from Fig. 6p and Fig. 6q (n = 5,6,3,3). Mean $\pm$ s.e.m. All statistical tests are two-tailed. n indicate biologically independent experiments except for Extended Data Fig. 4 in which n indicate independent samples and Extended Data Fig. 4n–q in which n indicate independent animals.

To broaden our investigation of nickase-based gene editors, we then included prime editing in our study. We first designed a panel of pegRNAs spanning *B2M* to induce its KO. Each pegRNA was also paired with a gRNA to explore a PE3 setup, generating a nick on both DNA strands (Fig. 6a). No *B2M* modification was observed in K-562 cells for all pegRNAs tested except for pegRNA5, which induced 20% and 90% modified alleles when used without or with the cognate gRNA, respectively (Fig. 6b). We then tested the selected PE3 setup in mPB-derived HSPCs from six independent healthy donors and treated cells after 3 d of culture when expression of most genes belonging to the DNA mismatch repair (MMR) pathway, which may antagonize prime editing, becomes lower^24,31^ (Extended Data Fig. 4a,b). *B2M*-KO cells were 30% in the bulk culture and 35% in the most primitive compartment (Fig. 6c and Extended Data Fig. 4c) without detectable changes in composition of progenitor subsets (Fig. 6d). Molecular analysis revealed up to 60% modified *B2M* alleles, without detectable deletions spanning from one to the other nicking site by PCR (Extended Data Fig. 4d,e). Deep-sequencing analysis of the *B2M* target site showed an average 40% precise prime editing outcome and 4.5% with additional insertion of the first bases of the pegRNA scaffold or small deletions at either nicking site (Fig. 6e and Extended Data Fig. 4f).

Transcriptional analysis performed 24 h after PE3 treatment showed significant upregulation of genes related to IFN signaling (*IFI6* and *ISG15*), p53 activation (*CDKN1A* and *MDM2*) and unfolded protein response (*HSPA5* and *ATF3*; Extended Data Fig. 4g). Enrichment analysis confirmed activation of these pathways in PE3-treated cells compared to mock-electroporated HSPCs (Fig. 6f). To stringently compare Cas9 and PE3 side by side, we combined Cas9 nuclease with *B2M* exon 1 gRNA that contains the spacer sequence of pegRNA5 as the targeting region (Extended Data Fig. 4h) and reached 80% and 50% allele modification, respectively, by the two systems (Fig. 6g). Cas9-treated, but not PE3-treated, HSPCs showed a trend toward lower clonogenic capacity than mock-electroporated cells, indicating a stronger impact of Cas9 than PE3 on HSPCs (Fig. 6h). Screening of around 140 randomly picked single colonies revealed the occurrence of large deletions after both treatments, although to a lower extent with PE3 than with Cas9 (Fig. 6i and Extended Data Fig. 4i). We next interrogated PE3- and Cas9-edited HSPCs for activation of selected IFN response and p53 pathway genes and found a slight induction of the former pathway genes in PE3 samples and an upregulation of the latter pathway genes in both treatments to a lower extent in PE3-edited samples than in Cas9-edited samples, consistent with the above findings (Fig. 6j,k and Extended Data Fig. 4j). We found selective activation of the proapoptotic isoform of *TP73* after prime editing, which was completely absent when performing either Cas9 or base editing (Fig. 6l and Extended Data Fig. 4k,l). Moreover, we found a mild but specific activation of *MT2A* in PE3-edited samples, supporting the activation of apoptotic responses (Fig. 6m). Overexpression of *TP73* by PE-edited samples occurred in the presence and absence of pegRNA in all six mPB HSPC donors tested (Fig. 6n) but was absent for a catalytically inactive RT fused with the nickase. However, a catalytically active RT fused with dCas9 failed to induce a similar response, showing that RT activity is necessary but not sufficient to induce proapoptotic *TP73* transcription. Together, these results suggest that *TP73* induction requires both RT activity and a concomitant nick at its DNA binding site, whether mediated by the gRNA or pegRNA. Notably, the use of the PE3max strategy, which improved prime editing efficiency by approximately 1.5-fold (Extended Data Fig. 4m), prevented induction of *TP73* but not *MT2A* (Fig. 6o).

Prime-edited HSPCs engrafted and persisted long term in xenotransplanted mice, maintaining >50% editing efficiency in PB and hematopoietic organs (Fig. 6p,q and Extended Data Fig. 4n,o) with no skewing of lineage compositions (Extended Data Fig. 4p,q). The graft size of PE3-treated cells was reduced compared to mock-electroporated cells, in particular at early times, conceivably due to a detrimental impact of the cellular responses described above on short-term repopulating progenitors. Deep-sequencing analysis of the *B2M* target site on PB and BM cells revealed an average of 42% precise and 5% imprecise prime editing outcomes (Fig. 6r). Prime-edited HSPCs were able to engraft in secondary recipients, maintaining >50% efficiency in PB and hematopoietic organs (Fig. 6s–v and Extended Data Fig. 4p,q).

These data show that prime editing may reach substantial efficiency in long-term repopulating HSPCs and thus potentially broaden applications of genome editing to include transversion and other changes in the target sequence, with the current caveat of selecting an effective pegRNA. As also shown for BE, PE can still induce DNA DSBs and deletions at the target site, albeit to a lower extent than nuclease-based editing, and does not escape cellular sensing of its unique machinery comprising a nickase and RT.

## Discussion

Here, we investigated the application of state-of-the-art nickase-based editing systems to human HSPCs and found that, compared to conventional Cas nuclease-based editing, the CBE, ABE and PE decrease, but do not abolish, the occurrence of DNA DSBs at their genomic targeted sites, exposing cells to the potential genotoxic effects of deletions and translocations. The rate of these adverse outcomes was higher for the CBE and substantially aggravated when the endogenous BER pathway was not adequately inhibited, either because of higher activity in specific cellular stages or suboptimal expression of the editor. All systems induced detrimental transcriptional responses in the treated cells that negatively impacted editing efficiency (for CBE) and/or clonogenic and repopulation capacity (for PE), albeit to a lesser extent than conventional nuclease-based editing. While these findings instructed strategies to minimize adverse outcomes and reach nearly exhaustive nickase-based editing in long-term repopulating HSPCs, genome-wide analyses uncovered a global impact of BE exposure on the mutational landscape of treated HSPCs, raising concerns for the occurrence of gRNA-independent deamination and unintended engagement of error-prone endogenous DNA repair pathways.

The differences in efficiency and cellular responses observed for nuclease versus nickase editing and among the different BEs and PEs, even when targeted to the same locus, likely reflect the different biochemical reactions captured to install the edits and the type of genome configuration and cellular environment where the source enzyme naturally evolved. Indeed, the higher efficiency of ABE may reflect the absence of an endogenous antagonizing pathway in human cells, which instead is present and hinders fixation of the intended mutation both for CBEs and PEs. However, it is possible that the higher precision of the ABE than the CBE may reflect a lower processivity and binding affinity of the former editor for eukaryotic DNA than its natural activity on bacterial RNA^32^. Whereas most of our findings were conserved across multiple target sites and HSPC sources, BEs and PEs are constantly evolving^13^, leading to superior efficiencies, sometimes at the cost of targeting specificity. Continuous engineering guided by the type of experimental findings reported here and the rationale design of next-generation editing systems may allow further performance improvement of these transformative tools^32,33^. For instance, delivery of editor protein instead of mRNA might avoid responses due to mRNA sensing and possibly mitigate unwanted editing outcomes, despite that their production and purification remains challenging to afford and standardize.

Activation of IFN responses was observed after delivery of long and complex mRNA structures and may contribute to lower engraftment of edited cells, in particular for long-term repopulating progenitors. This hurdle was nearly completely overcome by optimizing RNA design to increase yield, purity and stability. As we previously reported, p53 pathway activation consequent to DNA DSBs strongly impacted the size and clonality of the human graft in transplanted mice^6^. Although this response was well evident for Cas nuclease editing, both base and prime editing induced detectable activation of p53 target genes, where PE and BE4max were higher than ABE8.20-m, resulting in lower engraftment than control-treated samples. The induction of p53 target genes observed for each system correlated to some extent with the proportion of indels and large deletions found at the target site, formally proving induction of DNA DSBs. The DNA damage response was abrogated when transiently inhibiting p53, albeit at the cost of slightly reduced efficiency for all systems, possibly due to competition between editors and p53 inhibitor mRNA entry and translation. Yet, the increased proportion of indels at the BE4max target site suggests reduced purging of clones experiencing higher DNA damage burden and discourages from adopting p53 inhibition in this context.

PE3 showed specific induction of the proapoptotic transcript *TP73*, which might be a consequence of the formation of on-target editing intermediates, induced by the concurrent DNA nick and local RT, especially when not rapidly resolved, and/or MMR activation. These hypotheses are respectively in line with the reported induction of *TP73* after pharmacological topoisomerase inhibition in eukaryotic cells^34^ and with TP73-dependent apoptosis triggered by MMR^35^.

**Extended Data Fig. 5:**
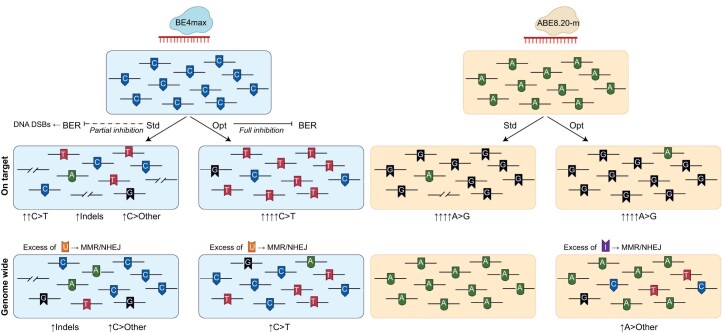
Schematic representation of BE mutagenesis on target and genome wide. Schematic representation of the possible molecular mechanisms leading to mutagenic events at the target sites and genome wide upon base editing.

Concerning the mechanisms underlying the generation of DNA DSBs, there may be common and specific factors for each editing system (Extended Data Fig. 5). Conversion of an SSB to a DSB after transit of a DNA replication fork is likely to be a shared mechanism among all nickase-based systems^36^. The higher fraction of alleles carrying deletions and the stronger propensity to generate translocations observed for the CBE likely reflect the involvement of BER by UG recruitment at uracil nucleotide residues and subsequent APEX1-dependent nick. If this repair process is not inhibited, the combination of APEX1- and BE-dependent nicks on the two opposite strands may result in a staggered DNA DSB at the target site, which may be eventually repaired by non-homologous/microhomology-mediated end joining. Supporting this explanation is the observation of larger proportions of alleles carrying deletions in BE4max-treated cells at day 1 than at day 3, when the BER machinery is upregulated. Similarly, the decrease in alleles carrying indels over time in the graft, when the output of long-term repopulating cells becomes prevalent, may correlate with the lower BER gene expression in primitive than in committed progenitors, a finding concordant with a previous report on mouse HSPCs^37^. While more robust expression of Cas9 by mRNA optimization increased the proportion of indels at the target, we observed the opposite result for BE4max, with fewer deletions and more stable and polyclonal edited cell grafts in vivo. This apparent paradox might be explained by more robust inhibition of UG activity by the UGI domains coupled to the BE.

The substantial amount of DNA DSBs in HSPCs emphasizes a concrete liability of nickase-based editing and CBE in particular, which were originally described as spared from potentially genotoxic events and thus defined as ‘DSB-free’, rather than ‘DSB-less’, systems. Although the frequencies of deletions and translocations were lower after nickase-based editing than after nuclease-based editing, these figures remain relevant considering that several hundred million HSPCs are treated and infused in clinical applications of HSPC gene therapy (>2 × 10^6^ CD34^+^ cells per kg)^38^. Hence, the potential occurrence and in vivo persistence of large genomic rearrangements should be considered in the preclinical and clinical risk assessment of base and prime editing and, even more stringently, when aiming for multiplex editing approaches. In the latter context, epigenome editing might eventually provide an intriguing alternative for targeted gene KO^39,40^.

It is conceivable that intrinsic features of the locus may influence the efficiency and precision of base and prime editing. For instance, the presence of multiple editable nucleotides in the target sequence (as in the case of BE4max for the *B2M* exon 2 target site) may cause tandem deaminations and affect the type and kinetics of repair, leading to different proportions of byproducts in the outcome. Moreover, bystander editing may limit the use of base editing when aiming to correct specific disease-causing mutations. Similarly, the challenge in designing an efficient pegRNA, as reported here, highlights a potential hurdle of the current PE system that may be overcome by next-generation molecules and trained algorithms^41,42^.

One of the most challenging aspects of investigating the specificity of emerging editing systems combining a dead or nCas domain for tethering the editor to the intended target with a constitutively active enzyme is the possibility of gRNA-independent global activity of the latter on the genome. Such events may escape detection when interrogating complex mixtures of treated cells as bulk in vitro cultures or highly polyclonal grafts because of dilution and lack of recurrence in the experimental context. However, analysis of samples comprising the expanded outgrowth of a known or predicted small number of clones might help uncover an altered frequency or distribution of variants associated with specific treatments, as shown here for the clonally shrunken graft of BE4max-edited cells or pools of in vitro colonies formed by edited cells. Moreover, the engagement of different DNA repair pathways and genomic surveillance mechanisms by multiple concurrent DNA lesions may contribute to alter the mutational landscape and purge cells accruing excess mutational load and/or DNA adducts that cannot be processed.

In the case of BE4max, the transient overexpression of UGI and consequent inhibition of UG might impair the processing of spontaneous and induced cytidine deaminations, preventing initiation of endogenous BER and leading to engagement of the less faithful MMR or non-homologous end joining (Extended Data Fig. 5), which may allow incorporation of transversions and trigger the DNA damage response and apoptosis^43,44^. A broad mutation pattern is naturally installed by cytidine deaminases during somatic hypermutation, when MMR may interfere with BER because of excess U•G mismatches^45^. Of note, CBEs have also been previously reported to occasionally install transversions at the target site, with variable frequencies depending on the loci and the cell types^19,28^, despite the underlying mechanisms remaining unclear. In addition, the relatively high frequency of DNA DSBs induced at gRNA-dependent target sites of BE4max also causes p53 activation, leading to loss of engrafting capacity, and may thus purge the cells that have experienced highest exposure to the BE. Both processes may result in depletion of C>T/G>A transitions and provide an indirect readout of interference with normal DNA repair processes. Notably, when induction of DNA DSBs was alleviated by improved BE expression, the expected increase in these transitions appeared to emerge (see Fig. 5r). In the case of ABE8.20-m, where no specific excision and repair pathway exists for DNA-embedded inosines, one can expect engagement of MMR or non-homologous end joining in the absence of a concurrent DNA nick on the opposite strand, as it occurs instead at the target site (Extended Data Fig. 5). Error-prone repair may thus emerge as BE expression is increased, as noted in our experiments (compare Figs. 4k and 5r).

We acknowledge that the actual mechanisms underlying the observed skewed proportions of SNVs after exposure to the different BEs reported here remain speculative and incompletely understood. However, as we reproduced these observations using orthogonal analyses, we think it appropriate to report them as they raise concerns for a potential genotoxic impact of these systems that until now has been unappreciated. As experimental conditions might alleviate or aggravate such an impact, as shown here when treating cells at different culture times or using BE expression constructs with different efficiency, further studies to investigate the mechanism(s) underlying any global impact of BEs and to devise strategies circumventing them are recommended for a comprehensive assessment of the risk benefit associated with these technologies. Overall, the blueprint and set of metrics described in this study will instruct careful and comprehensive evaluation of emerging editing tools and strategies, which would be helpful in fundamental research and toward clinical translation.

## Methods

### Plasmids

The pCMV_BE4max plasmid was a gift from D. Liu (Addgene plasmid 112093; https://www.addgene.org/112093/; http://n2t.net/addgene:112093). The ABE8.20-m plasmid was a gift from N. Gaudelli (Addgene plasmid 136300; https://www.addgene.org/136300/). The pCMV-PE2 plasmid was a gift from D. Liu (Addgene plasmid 132775; https://www.addgene.org/132775/). pCMV-PEmax was a gift from D. Liu (Addgene plasmid 174820; https://www.addgene.org/174820/; RRID:Addgene_174820). The Cas9_WPRE-polyA and dCas9_WPRE-polyA plasmids were gifts from A. Lombardo (SR-Tiget). nCas9, dCas9-RT and nCas9-dRT were obtained by mutagenesis from Cas9_WPRE-polyA and pCMV-PE2 plasmids, respectively. All plasmids carried the T7 promoter downstream of the CMV promoter. For the generation of constructs for standard mRNA in vitro transcription, the WPRE followed by a poly(A) sequence was subcloned into the above-mentioned plasmids downstream of the coding sequence. For the generation of constructs for optimized mRNA in vitro transcription, the following sequences were subcloned in the standard plasmid in place of the T7 sequence for anti-reverse cap analog capping and the 5′ UTR: CapAG–eIF4G aptamer (GACTCACTATTTGTTTTCGCGCCCAGTTGCAAAAAGTGTCG), Kozak sequence (CCACC) and start codon (ATG), as previously described^46^. The pU6-pegRNA-GG-acceptor was a gift from D. Liu (Addgene plasmid 132777; https://www.addgene.org/132777/). Plasmids expressing gRNAs targeting *B2M* were cloned in a pU6 plasmid using annealed oligonucleotides described in Supplementary Table 2. The *B2M* gRNA for base and Cas9 editing was previously described in Gaudelli et al.^20^. pegRNAs targeting *B2M* were designed with pegFinder (http://pegfinder.sidichenlab.org/)^47^ using the default parameters. pegRNAs were subcloned in the pU6-pegRNA-GG-acceptor using annealed oligonucleotides described in Supplementary Table 2.

### mRNA in vitro transcription

Standard and optimized plasmids were linearized with SpeI or PmeI (New England Biolabs) and purified by phenol–chloroform extraction. Different preparations of mRNAs for each editor were in vitro transcribed using the commercial 5× MEGAscript T7 kit (Thermo Fisher). Standard mRNAs were capped with 4.5 mM anti-reverse cap analog 3′-*O*-methyl-mG(5′) ppp(5′)G (New England Biolabs) mixed in a 1:5 ratio with dGTP nucleotides. Optimized mRNAs were capped with 8 mM CleanCapAG (Trilink)^46^. mRNAs were purified using an RNeasy Plus Mini kit (Qiagen). mRNAs were denaturated and resolved by capillary electrophoresis on a 4200 TapeStation System (Agilent), according to the manufacturer’s instructions, to assess quality and integrity. mRNAs were purified by high-performance liquid chromatography (ADS BIOTEC WAVE System) and concentrated with Amicon Ultra-15 (30,000-Da molecular weight cutoff) tubes (Millipore). mRNA productions were then aliquoted and stored at −80 °C. Reproducible results were obtained in replicate experiments using different preparations of the same editor mRNA.

### Cell lines and primary cell culture

B lymphoblastoid cells were cultured in RPMI 1640 medium (Corning) supplemented with 10% heat-inactivated fetal bovine serum (FBS; Euroclone), 100 IU ml^−1^ penicillin, 100 µg ml^−1^ streptomycin and 2% glutamine.

K-562 cells (ATCC) were cultured in Iscove’s modified Dulbecco’s medium (Corning) supplemented with 10% heat-inactivated FBS, 100 IU ml^−1^ penicillin, 100 µg ml^−1^ streptomycin and 2% glutamine.

Human primary T cells were isolated from PB mononuclear cells (PBMCs) from healthy donors that were freshly purified from buffy coats with SepMate PBMC isolation tubes (StemCell Technologies), according to manufacturer’s instructions. Buffy coats were obtained in accordance with the Declaration of Helsinki as anonymized residues of blood donations and were used upon signature of specific institutional informed consent for blood product donation by healthy blood donors. CD3^+^ T cells were stimulated using magnetic beads (1:3 cell:bead ratio) conjugated with antibodies to CD3 and CD28 (Dynabeads human T-activator CD3/CD28, Thermo Fisher). Cells were maintained in Iscove’s modified Dulbecco’s medium (Corning) supplemented with 10% heat-inactivated FBS, 100 IU ml^−1^ penicillin, 100 µg ml^−1^ streptomycin, 2% glutamine, 5 ng ml^−1^ human interleukin-7 (hIL-7; PreproTech) and 5 ng ml^−1^ hIL-15 (PreproTech). Dynabeads were removed after 6 d of culture.

CB CD34^+^ HSPCs were purchased from Lonza according to the TIGET-HPCT protocol approved by the Ospedale San Raffaele (OSR) Ethical Committee and seeded at a concentration of 5 × 10^5^ cells per ml in serum-free StemSpan SFEM (StemCell Technologies) supplemented with 100 IU ml^−1^ penicillin, 100 μg ml^−1^ streptomycin, 2% glutamine, 100 ng ml^−1^ human stem cell factor (PeproTech), 100 ng ml^−1^ human Flt3-L (PeproTech), 20 ng ml^−1^ human thrombopoietin (PeproTech), 20 ng ml^−1^ hIL-6 (PeproTech), 10 μM 16,16-dimethyl prostaglandin E_2_ (added at the beginning of the culture; Cayman), 1 μM SR1 (Biovision) and 50 nM UM171 (StemCell Technologies).

Granulocyte colony-stimulating factor (G-CSF) or G-CSF + plerixafor mPB CD34^+^ HSPCs were purified in-house with the CliniMACS CD34 Reagent system (Miltenyi Biotec) from Mobilized Leukopak (AllCells) according to the TIGET-HPCT protocol approved by the OSR Ethical Committee and following the manufacturer’s instructions. HSPCs were seeded at a concentration of 5 × 10^5^ cells per ml in serum-free StemSpan SFEM supplemented with 100 IU ml^−1^ penicillin, 100 μg ml^−1^ streptomycin, 2% glutamine, 300 ng ml^−1^ human stem cell factor, 300 ng ml^−1^ human Flt3-L, 100 ng ml^−1^ human thrombopoietin, 10 μM 16,16-dimethyl prostaglandin E_2_ (added at the beginning of the culture), 1 μM SR1 and 35 nM UM171.

All cells were cultured in a humidified atmosphere at 5% CO_2_ and 37 °C.

### Gene editing of cell lines and analyses

For each condition, 3.0 × 10^5^ cells were washed with ten volumes of Dulbecco’s phosphate-buffered saline (DPBS; Corning) without Ca^2+^ and Mg^2+^ and electroporated using an SF Cell Line 4D-Nucleofector X kit (Lonza). B lymphoblastoid cells were pulsed with program EW-113, and K-562 cells were pulsed with program FF-120. For base editing, B lymphoblastoid cells were electroporated with 0.5 μg of *B2M* gRNA plasmid and either 2.0 μg of BE plasmid (Addgene) or 4.0 μg of standard BE mRNAs unless otherwise specified. For prime editing, K-562 cells were electroporated with 0.25 μg of pegRNA plasmids and 1 μg of PE2 plasmid. For PE3 conditions, 0.25 μg of respective gRNA plasmids was added to the electroporation mixture. Cells were cultured for 7 d, analyzed by flow cytometry and collected for genomic (gDNA) extraction and subsequent molecular analysis.

### Gene editing of human T cells and analyses

For each condition, 5.0 × 10^5^–1.0 × 10^6^ human T cells were washed with ten volumes of DPBS without Ca^2+^ and Mg^2+^ and electroporated using a P3 Primary Cell 4D-Nucleofector X kit (Lonza) and program DS-130. Cells were electroporated with 75 pM *B2M* gRNA (Synthego) and 3.0 μg of standard mRNAs unless otherwise specified. Cells were cultured for 7 d, analyzed by flow cytometry and collected for gDNA extraction and subsequent molecular analysis. The *B2M* gRNA spacer sequence for base and Cas9 editing is shown in Supplementary Table 3.

### Gene editing of human HSPCs and analyses

For each condition, from 2.0 × 10^5^ to 7.5 × 10^5^ CB/mPB-derived HSPCs were washed with ten volumes of DPBS without Ca^2+^ and Mg^2+^ and electroporated using a P3 Primary Cell 4D-Nucleofector X kit (Lonza) and program EO-100 after either 1 or 3 d of culture, as indicated. For base and Cas9 editing, HSPCs were electroporated with 75 pmol of gRNA (*B2M* exon 2, *AAVS1*, *B2M* exon 1, *BLC11a*, *CCR5* and *IL2RG*) and 7.5 μg of standard mRNAs, unless otherwise specified, or 3.5 μg (low dose) or 7.5 μg (high dose) of optimized mRNAs. For PE, HSPCs were electroporated with 186 pmol of *B2M* pegRNA5, 75 pmol of *B2M* exon 1 gRNA and 7.5 μg of optimized PE mRNA. Seven days after electroporation, HSPCs were collected for flow cytometry analyses and gDNA extraction for molecular analysis. Colony-forming cell (CFC) assays were performed 24 h after editing by plating 400–800 cells in methylcellulose-based medium (MethoCult H4434, StemCell Technologies) supplemented with 100 IU ml^−1^ penicillin and 100 µg ml^−1^ streptomycin. Three technical replicates were performed for each condition. Two weeks after plating, colonies were counted and eventually picked for molecular analysis or exome sequencing. The gRNA spacer sequences for base, Cas9 and prime editing are shown in Supplementary Table 3.

### Mice

All experiments and procedures involving animals were performed with the approval of the Animal Care and Use Committee of the San Raffaele Hospital (IACUC 1206) and were authorized by the Italian Ministry of Health and local authorities accordingly to Italian law. NOD-*scid*-*Il2rg*^−/−^ (NSG) female mice (The Jackson Laboratory) were held under specific pathogen-free conditions.

### CD34^+^ HSPC xenotransplantation experiments in NSG mice

For xenotransplantation of CB and G-CSF mPB HSPCs, the outgrowths of 5.0 × 10^4^ to 1.25 × 10^5^ and 5.0 × 10^5^ to 7.5 × 10^5^ HSPCs, respectively, at the start of the culture (*t*_0_ equivalent) were injected intravenously 24 h after editing into sublethally irradiated NSG mice (180–200 cGy). Matched numbers of HSPCs were seeded at day 0 of culture for each experimental group to transplant the same number of culture-initiating HSPCs in each mouse. Mice were randomly distributed to each experimental group. Human CD45^+^ cell engraftment and the presence of edited cells were monitored by serial collection of blood (approximately every 2 to 3 weeks) from the retroorbital plexus, and, at the end of the experiment (15–16 weeks after transplantation), BM and SPL were collected for end point analyses, including florescence-activated cell sorting of hematopoietic lineages in some experiments.

Secondary transplantation in NSG mice was performed by transplanting bead-purified (Miltenyi Biotec) human CD34^+^ cells from the BM of primary recipients. CD34^+^ cells from all mice of each experimental group were pooled and split in recipients according to the input number of cells.

### Flow cytometry

Immunophenotypic analyses were performed by flow cytometry using Canto II (BD Pharmingen). From 5.0 × 10^4^ to 2.0 × 10^5^ cells either from culture or mouse-derived samples were analyzed. Cells were stained for 15 min at 4 °C with antibodies listed in Supplementary Table 4 in a final volume of 100 μl and were washed with DPBS + 2% heat-inactivated FBS. Single-stained and fluorescence-minus-one-stained cells were used as controls. The Live/Dead Fixable Dead Cell Stain kit (Thermo Fisher) or 7-aminoactinomycin D (7AAD; Sigma-Aldrich) was included during sample preparation, according to the manufacturer’s instructions, to identify dead cells. Analysis of apoptosis was performed on T cells 1 d after electroporation using Pacific Blue-conjugated Annexin V (Biolegend) and an Apoptosis Detection kit with 7AAD (BD Pharmingen) according to the manufacturers’ instructions. Percentages of live (7AAD^−^Annexin V^−^), early apoptotic (7AAD^−^Annexin V^+^), late apoptotic (7AAD^+^Annexin V^+^) and necrotic (7AAD^+^Annexin V^−^) cells were reported. Cell sorting was performed on a BD FACSAria Fusion (BD Biosciences) with BD FACS Diva software v8.0.1 and equipped with four lasers: blue (488 nm), yellow/green (561 nm), red (640 nm) and violet (405 nm). Cells were sorted with an 85-µm nozzle. Sheath fluid pressure was set at 45 psi. A highly pure sorting modality (four-way purity sorting) was chosen. Cell sorting was performed on a MoFlo Astrios EQ (Beckman Coulter) with Summit software and equipped with four lasers: blue (488 nm), yellow/green (561 nm), red (640 nm) and violet (405 nm). Cells were sorted with a 100-µm nozzle. Sheath fluid pressure was set at 25 psi. A highly pure sorting modality (purify-1 sorting) was chosen. Sorted cells were collected in 1.5-ml Eppendorf tubes containing 500 μl of DPBS. Gating strategies are provided in Supplementary Figs. 3 and 4. Data were analyzed with FCS Express 7 Flow.

### Molecular analyses

For molecular analyses, gDNA was isolated with a QIAamp DNA Micro kit (Qiagen) according to the manufacturer’s instructions. Extraction of gDNA from colonies in CFC assays was performed with QuickExtract (Epicentre) according to the manufacturer’s instructions. When specified, BE and Cas9 efficiencies were measured by PCR amplification at the target locus, followed by amplicon Sanger sequencing (Eurofins Scientific), whose results were then analyzed by EditR software (http://baseeditr.com)^48^ using default parameters or by TIDE software (https://tide.nki.nl/)^49^. When specified, PE efficiencies were measured by PCR amplification at the target locus, followed by amplicon Sanger sequencing (Eurofins Scientific), whose results were then analyzed by EditR software. To adapt EditR for *B2M* prime editing, we used as input the sequence TGGCCTTAGCTGTGCTCGC and selected the reverse complement orientation option.

For droplet digital PCR (ddPCR) analyses, 5–50 ng of gDNA was analyzed using the QX200 Droplet Digital PCR System (Bio-Rad) according to the manufacturer’s instructions. Primers and probes for vector copy number (VCN) were previously reported^50^. Primers and probes to detect large *B2M* deletions were designed upstream and downstream of the DNA SSB of base and prime editing or of the Cas9 DSB, as shown in Extended Data Figs. 1k and 4i. Human *TTC5* (Bio-Rad) or *GAPDH* (Bio-Rad) assays were used for normalization. Copy numbers for both VCN and deletion analyses were calculated with the following formula: (number of LV/*B2M*^+^ droplets/number of normalizer^+^ droplets) × 2.

For translocation analyses, 100 ng of gDNA was amplified. DNA amplicons were resolved by capillary electrophoresis on a 4200 TapeStation (Agilent) according to the manufacturer’s instructions.

For gene expression analyses, total RNA was extracted using an RNeasy Plus Micro kit (Qiagen) according to the manufacturer’s instructions. DNase treatment was performed using an RNase-free DNAse set (Qiagen). cDNA was synthesized using a SuperScript VILO IV cDNA Synthesis kit (Thermo Fisher) with EzDNAse treatment. Two nanograms of cDNA was then used for gene expression analysis by ddPCR. Relative expression of each target gene was first normalized to *HPRT* and then represented as fold changes relative to untreated cells. Primers, probes and gene expression assays are listed in Supplementary Table 2. ddPCR data were analyzed with QuantaSoft Software v1.7.4 (Bio-Rad). Thermal cycling protocols are listed in Supplementary Table 5.

### Deep-sequencing and bioinformatic analyses

PCR amplicons for individual samples were generated by nested PCR using primers listed in Supplementary Table 2 starting from >50 to 100 ng of purified gDNA. For *B2M* exon 2, *AAVS1*, *B2M* exon 1, *BCL11A* and *IL2RG*, the first PCR step was performed with GoTaq G2 DNA Polymerase (Promega) according to manufacturer’s instructions. The second PCR step was performed using the same reagents of the first step and 5 μl of the PCR. For *CCR5* base editing, a preamplification step followed by first and second PCR was performed with GoTaq G2 DNA Polymerase (Promega) according to manufacturer’s instructions. Thermal cycling protocols are listed in Supplementary Table 5. Primers used for the second PCR step contained P5/P7 sequences, i5/i7 Illumina tags to allow multiplexed sequencing and R1/R2 primer binding sites. The PCR amplicon from each sample was separately purified by using a QIAquick PCR Purification kit (Qiagen). Concentration and quality of amplicons were assessed by using a QuantiFluor ONE dsDNA system and 4200 Tapestation system (Agilent). Amplicons from up to 49 differently tagged samples were multiplexed at equimolar ratios and run by the San Raffaele Center for Omic Sciences (COSR) using 1 × 150 bp paired-end MiSeq (Illumina).

Sequencing data were analyzed with CRISPResso2 (v2.2.8), which enables the detection of small variants in gene editing experiments^51^. More precisely, for each sample, input reads were trimmed (CRISPResso2 options: –trim_sequences –trimmomatic_command trimmomatic –trimmomatic_options_string‘ILLUMINACLIP:TruSeq3-PE-2.fa:2:30:10 MINLEN:100’) to remove low-quality positions (score of <30) and to remove Illumina adapters, keeping only trimmed sequences longer than 100 bp to ensure the full coverage of the region of interest. Sequences were then mapped to the input amplicon reference, and the quantification window was set to 1 bp around the cut site, as identified by providing the gRNA sequence. Computed alleles were quantified by measuring the number of reads and their relative abundances based on total read counts. Moreover, depending on the type of experiment (that is, base or prime editing), different input options were given to CRISPResso2 to perform the specific analyses. For base editing analyses, both the targeted and the edited nucleotides were provided (CRISPResso2 options:–base_editor_output–conversion_nuc_from T/G–conversion_nuc_to C/A) to measure the frequency of the expected nucleotide substitutions for the specific BE. For the prime editing analyses, sequences for the pegRNA spacer, extension and scaffold as well as for the additional nicking gRNA and the reference amplicon were provided as input to identify and quantify precise prime editing (that is, carrying only the expected edit), imprecise prime editing (that is, containing the prime editing and additional modifications, such as partial scaffold incorporation and indels) and other events. Finally, CRISPResso2 output alleles were postprocessed by correcting all the mismatch positions outside the quantification window and requantifying the total read counts and consequently the corresponding relative abundances.

### HSPC transduction with BAR LV for clonal tracking

The transfer vector construct for the BAR LV will be described in detail elsewhere. The LV was produced as described in Soldi et al.^50^. Clonal tracking was performed on a pool of HSPCs derived from four CB donors. One day after thawing, HSPCs were transduced at a concentration of 1 × 10^6^ cells per ml with the BAR LV using a multiplicity of infection of 30 transducing units per ml. HSPCs were washed with ten volumes of DPBS without Ca^2+^ and Mg^2+^ 24 h later and then (24 h after washing) treated for editing (or mock electroporation) and transplanted as described above.

### BAR-seq clonal tracking

PCR amplicons for individual samples retrieved from sorted hematopoietic organs and lineages were generated by nested PCR using primers listed in Supplementary Table 2 and starting from >50 to 100 ng of purified gDNA, as previously described^52^. In detail, the first PCR step was performed with GoTaq G2 DNA Polymerase (Promega) according to the manufacturer’s instructions. The second PCR step was performed using the same reagents as the first step and 5 μl of the PCR product. Thermal cycling protocols are listed in Supplementary Table 5. Primers used for the second PCR step contained P5/P7 sequences, i5/i7 Illumina tags to allow multiplexed sequencing and R1/R2 primer binding sites. The PCR amplicon from each sample was separately purified by QIAquick PCR Purification kit (Qiagen). Concentration and quality of amplicons were assessed by using a QuantiFluor ONE dsDNA system and 4200 Tapestation system (Agilent). Amplicons from up to 85 differently tagged samples were multiplexed at equimolar ratios and run by the San Raffaele COSR using 2 × 75 bp paired-end NextSeq (Illumina).

Sequencing data were analyzed with the BAR-Seq2 pipeline (https://bitbucket.org/bereste/bar-seq2). In detail, input reads were preprocessed to trim low-quality bases and keep sequences of a length of ≥50 bp (options: -m 50 -q 30) to ensure the proper amplicon structure within each read. BARs were then extracted using TagDust and corrected using a community-based strategy on a graph built on the sequence similarity (edit distance of ≤2). Resulting BARs were quantified based on their abundances (number of supporting reads) and filtered, keeping only those with a minimum count equal to 2. The numbers of clones were calculated for each sample by normalizing the number of unique BARs by the sample VCN. SRC frequency (1 of ‘x’) was calculated by dividing the HSPC *t*_0_ equivalent by the number of unique BARs retrieved in the long-term human graft in the BM.

### Total RNA-seq library preparation and bioinformatic analysis

Whole-transcriptomic analysis was performed on a pool of HSPCs derived from six CB donors. All conditions were performed in triplicate. Total RNA was purified 24 h after editing using an RNeasy Micro kit (Qiagen). DNase treatment was performed using an RNase-free DNAse set (Qiagen) according to the manufacturer’s instructions. RNA was quantified with a Qubit 2.0 fluorometer (Thermo Fisher), and quality was assessed with a 2100 Agilent Bioanalyzer (Agilent Technologies). Minimum quality was defined as an RNA integrity number of >8. Three hundred nanograms of total RNA was used for library preparation with a TruSeq Stranded mRNA kit (Illumina) and sequenced on a NextSeq 500 High 75 (Illumina) by the San Raffaele COSR or Genewiz (Azenta Life Sciences).

Preprocessing of the input sequences was done with FastQC (v0.11.6) to assess read quality and with Trimmomatic to remove low-quality sequences. Reads were then aligned to the human genome assembly (GRCh38) using STAR software (v2.7.6a) with standard parameters, and abundances were calculated using the Subread featureCounts function (v2.0.1). Differential gene expression analysis was performed using the R/Bioconductor package DESeq2 (v1.30.0), normalizing for library size using DESeq2’s median of ratios. *P* values were corrected using FDR, and genes with an FDR of <0.05 were considered differentially expressed. Postanalyses on differential gene expression results were performed with the R/Bioconductor package ClusterProfiler (v4.7.1) using the Hallmark collection from MSigDB as the reference database. Visualization of the (spliced) alignments of the *TP73* gene was done with Integrative Genomes Viewer (IGV v2.8.0).

### gRNA-independent off-target evaluation of transcriptomes

Variant calling on RNA-seq base editing data was performed exploiting different tools similar to Li et al.^53^ and Rees et al.^54^. In detail, reads from replicates of each condition were pulled together, downsampled to 120 million and aligned to the human genome assembly (GRCh38) using STAR (v2.7.6a). Following the GATK ‘Best Practice Workflows’, as reported in Gaudelli et al.^20^, duplicates were then marked using Picard (v2.25.6) MarkDuplicates and GATK (v4.2.0) SplitNCigarReads to split reads containing N. Variants were then called using three different tools, namely, HaplotypeCaller (with options -min-base-quality-score 20, -dont-use-soft-clipped-bases and -standard-min-confidence-threshold-for-calling 20), Mutect2 (in tumor-only mode, with options -disable-read-filter MateOnSameContigOrNoMappedMateReadFilter) and FreeBayes (v1.3.5). Nucleotide composition of each position was also assessed using REDItools (https://github.com/tflati/reditools2.0) on each sample, discarding all the positions with coverage lower than 20 and base quality lower than 30 to avoid errors due to low sampling. Next, variants called by each tool in the untreated controls were filtered out in the treated samples to enrich for private mutations. This procedure retained only variants in high-quality genomic positions in both treated and untreated samples, for which the untreated sample showed ≥99% of reads supporting the reference, non-mutant base at the position of the mutation (based on REDItools). The final lists of variants for each sample were made by those called by all tools and that passed the filtering procedure (intersection).

### WES for the detection of gRNA-independent DNA off targets

For WES in Fig. 4, CB-derived HSPCs were edited as described above for the clonal tracking experiment and collected 7 d after the procedure to perform 500× WES of the in vitro bulk population. Cells from the same treatments were infused 1 d after treatment in NSG mice, and live human CD45^+^ cells from BM were retrieved 16 weeks after infusion for 500× WES. For mock-electroporated mice, live human CD45^+^ cells were sorted, gDNA was extracted, and sequencing was performed as described below. For Cas9 and BE4max mice, β_2_M^+^ (~50 and 65%, respectively) and β_2_M^–^ (50 and 35%, respectively) fractions were collected; for ABE8.20-m mice, only the β_2_M^−^ fraction was collected as it represented ~100% of the human graft. For Cas9 and ABE8.20-m, the β_2_M^−^ fractions were sequenced as described below; for BE4max, both β_2_M^+^ and β_2_M^−^ fractions were sequenced as described below. For WES in Fig. 5n–p, mPB-derived HSPCs were edited and collected 7 d after the procedure to perform 500× WES of the in vitro bulk population. Cells from the same treatments were infused 1 d after treatment in NSG mice, and live human CD45^+^ cells from BM were retrieved 16 weeks after infusion for 500× WES. For mock-electroporated mice, live human CD45^+^ cells were sorted, gDNA was extracted, and sequencing was performed as described below. For BE4max optimized and ABE8.20-m standard mice, β_2_M^−^ fractions were sorted and sequenced as described below. For WES in Fig. 5q–s, mPB-derived HSPCs from one donor were treated and plated 24 h later for CFC assays. The bulk mock-electroporated sample was also collected 24 h after editing and sequenced by 100× WES. Two weeks later, individual colonies were picked and screened for intended outcome, and six single colonies for each condition were pooled in equal gDNA amounts and sequenced by 100× WES.

All WES was performed by Genewiz (Azenta) using the Agilent SureSelect Human All Exon V7 kit and running on an Illumina NovaSeq (2 × 150 bp) with an estimated output of ~50 gigabases (500×) or ~10 gigabases (100×) per sample. WES data were analyzed following the GATK ‘Best Practice Workflows’ to identify variants in each sample. Briefly, the quality of the input reads was assessed using FastQC (v0.11.9), and low-quality bases were trimmed using trim-galore (v0.6.6). For samples retrieved from mice, a disambiguation was preformed to remove possible mouse contaminations. The latter operation was performed as described in Ahdesmäki et al.^55^, that is, by aligning sequences to human and mouse reference genomes and assigning each read to the organism showing the best alignment. Next, most abundant samples were randomly downsampled to 300 million, 230 million or 50 million reads according to the experiment using the Seqtk toolkit (v1.3) to avoid sample imbalance. Reads were then aligned to the human genome assembly (GRCh38) using BWA (v0.7.17). Alignments were processed to mark duplicates using Picard (v2.25.6) MarkDuplicates, and GATK (v4.2.0) BaseRecalibrator and ApplyBQSR were used to recalibrate base quality scores on dbSNP known sites. HaplotypeCaller in Genomic Variant Call Format (GVCF) mode was used to call variants in each sample, which were then combined using CombineGVCFs and genotyped with GenotypeGVCFs. Resulting variants were filtered using VariantFiltration based on their ‘QualityByDepth’ (that is, -filter-expression ‘QD < 2.0’) and overall coverage ‘DP’ (that is, -filter-expression ‘DP < 500’). To identify private variants belonging to each sample, additional filters were applied, that is, variants with low genotype quality (that is, GQ < 80) and low coverage (that is, DP < 50 and DP < 10) were removed. The mock-electroporated in vitro sample for each experiment was used as a germline reference, and its variants were filtered out from all other samples, as they were considered as present in the initial cell population and not induced by treatments. Moreover, for the BE4max group in WES in Fig. 4, variants of the samples positively sorted for *B2M* editing were merged with those of the negative samples for each mouse. A final refinement was performed to remove multiallelic variants (mainly involving repetitive sequences). Remaining variants were annotated using SnpEff (v5.0) on the canonical isoform from the GRCh38.p13.RefSeq reference database. Downstream analysis of the final variants was done by classifying them based on their type (insertion, deletion or SNV) and focusing on all SNVs to classify mutation events. Assessment of variants using a panel of cancer-related genes was performed based on variant annotations. An additional focus on low-frequency variants was performed for WES in Fig. 5q–s by using Mutect2 to call variants and then filtering those with coverage lower than 10. To enrich for variants private for each colony, including those installed by the treatment, we kept for the analysis only those in the expected range of variant allele frequency (that is, between 0.05 and 0.2), considering that each pool was composed of six colonies (12 alleles).

### Quantification and statistical analyses

The number of biologically independent samples, animals or experiments is indicated by *n*. For some experiments, different HSPC donors were pooled to account for donor-related variability and to reach the number of cells needed for the analyses. Data were summarized as median with IQR (or range) or mean ± s.e.m. depending on data distribution. Inferential techniques were applied in the presence of adequate sample sizes (*n* ≥ 5); otherwise, only descriptive statistics are reported. Two-tailed tests were performed throughout the study. The Mann–Whitney test was performed to compare two independent groups, while in the presence of more than two independent groups, the Kruskal–Wallis test followed by post hoc analysis using Dunn’s test was used. Moreover, the FDR approach was used to address the problem of multiplicity arising from simultaneously testing several hypotheses and to adjust *P* values. The Wilcoxon test was then applied in a matched-pairs experimental design. In the presence of repeated measures and more complex forms of dependency among observations, random-intercept LME models^56,57^ were fitted, specifying random-effect terms, even nested whenever necessary, on both experimental units and experiment ID codes, aiming at better capturing underlying structure and data heterogeneity. When fitting LME models, standard transformations (logarithm, square/cubic root and ordered quantile normalization) were applied to outcome variables to satisfy model assumptions. Post hoc analysis after LME was performed to evaluate all the pairwise comparisons of interest, fixing other variables included in the model at a chosen value. The FDR procedure was used as a method for adjusting *P* values. For the statistical analysis on long-range deletions, a mean ± 3 s.d. interval was calculated from the mock-electroporated group, which includes at least 89% of the data according to the theory for any shaped distribution. Other treatment groups were then compared, evaluating the proportion of observations falling below or above the lower limit of the derived interval using the Fisher’s exact test. Pairwise comparison of treatment groups was performed and adjusted for multiple comparisons using the FDR approach. For all analyses, the significance threshold was set at 0.05. Analyses were performed using GraphPad Prism v.9.4.0 (GraphPad) and R statistical software (version 4.1.2; https://cran.r-project.org/index.html). Detailed results of statistical analyses are shown Supplementary Table 6.

### Reporting summary

Further information on research design is available in the Nature Portfolio Reporting Summary linked to this article.

## Online content

Any methods, additional references, Nature Portfolio reporting summaries, source data, extended data, supplementary information, acknowledgements, peer review information; details of author contributions and competing interests; and statements of data and code availability are available at 10.1038/s41587-023-01915-4.

### Supplementary information

Supplementary Information Supplementary Figs. 1–4.

Reporting Summary

Supplementary Table 1–6 Supplementary Tables 1–6.

### Source data

Source Data Fig. 1 Uncropped scans of gels.

## Data Availability

All relevant data are included in the manuscript. The reagents described in this manuscript are available under a material transfer agreement with IRCCS Ospedale San Raffaele and Fondazione Telethon; requests for materials should be addressed to S.F. and L.N. BAR-seq, RNA-seq and targeted deep-sequencing data are deposited at Gene Expression Omnibus (accession number GSE218464)^58^, while WES data are deposited at European Nucleotide Archive with the following accession numbers: PRJEB58344 (in vivo experiment with standard mRNA constructs)^59^, PRJEB64207 (experiment on HSPC-derived colonies)^60^ and PRJEB64407 (in vivo experiment with standard and optimized mRNA constructs)^61^. All other raw data from the main figures have been deposited at Mendeley and are publicly available as of the date of publication^62^. Source data are provided with this paper.

## Code availability

The scripts for all bioinformatic analyses of RNA-seq, WES, BAR-seq and deep-sequencing data of the locus are freely available at http://www.bioinfotiget.it/gitlab/custom/fiumara_baseprimeed2022 (ref. ^63^).
