# Author Correction: Genotoxic effects of base and prime editing in human hematopoietic stem cells

**DOI:** 10.1038/s41587-024-02142-1

**Published:** 2024-01-25

**Authors:** Martina Fiumara, Samuele Ferrari, Attya Omer-Javed, Stefano Beretta, Luisa Albano, Daniele Canarutto, Angelica Varesi, Chiara Gaddoni, Chiara Brombin, Federica Cugnata, Erika Zonari, Matteo Maria Naldini, Matteo Barcella, Bernhard Gentner, Ivan Merelli, Luigi Naldini

**Affiliations:** 1 San Raffaele Telethon Institute for Gene Therapy, IRCCS San Raffaele Scientific Institute, Milan, Italy; 2 Vita-Salute San Raffaele University, Milan, Italy; 3 Pediatric Immunohematology Unit and BMT Program, IRCCS San Raffaele Scientific Institute, Milan, Italy; 4 University Center for Statistics in the Biomedical Sciences, Vita-Salute San Raffaele University, Milan, Italy; 5 National Research Council, Institute for Biomedical Technologies, Segrate, Italy

**Keywords:** Targeted gene repair, Stem-cell biotechnology, Haematopoietic stem cells

Correction to: *Nature Biotechnology* 10.1038/s41587-023-01915-4, published online 7 September 2023.

In the version of the article initially published, there were errors in Fig. 4d as the absolute numbers of variants referred to an earlier analysis. This has been amended, and the original and corrected Fig. 4d appear as Fig. 1 below. In the Methods section, in the second paragraph of the “WES for the detection of gRNA-independent DNA off targets” section, the text now reading “DP < 500” and “DP < 50 and DP < 10” originally read “DP < 50” and “DP < 100 stringent and DP < 10 relaxed, respectively”. These errors have been corrected in the HTML and PDF versions of the article.

**Fig. 1:**
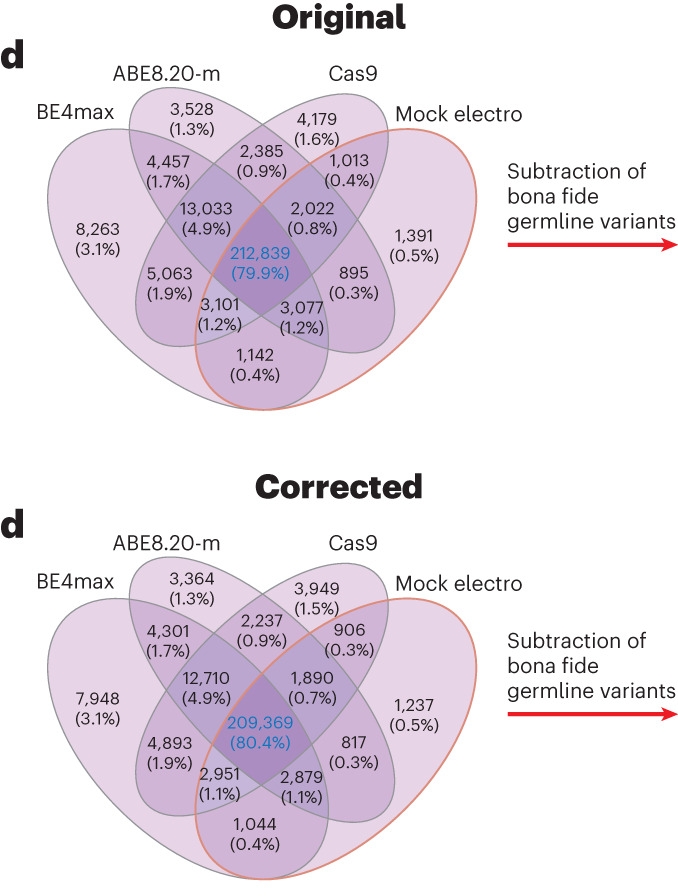
**Original and corrected Fig. 4d**.

