# Supplementary material for: Genotoxic effects of base and prime editing in human hematopoietic stem cells
Source: Nat Biotechnol. 2023 Sep 7;42(6):877–91. doi: 10.1038/s41587-023-01915-4 (PMC11180610; doi:10.1038/s41587-023-01915-4)
Supplement: Supplementary file 1 — Supplementary Figs. 1–4. [file 41587_2023_1915_MOESM1_ESM.pdf]

nature biotechnology

Article

https://doi.org/10.1038/s41587-023-01915-4

Genotoxic effects of base and prime editing in human hematopoietic stem cells

In the format provided by the authors and unedited

Supplementary Figures

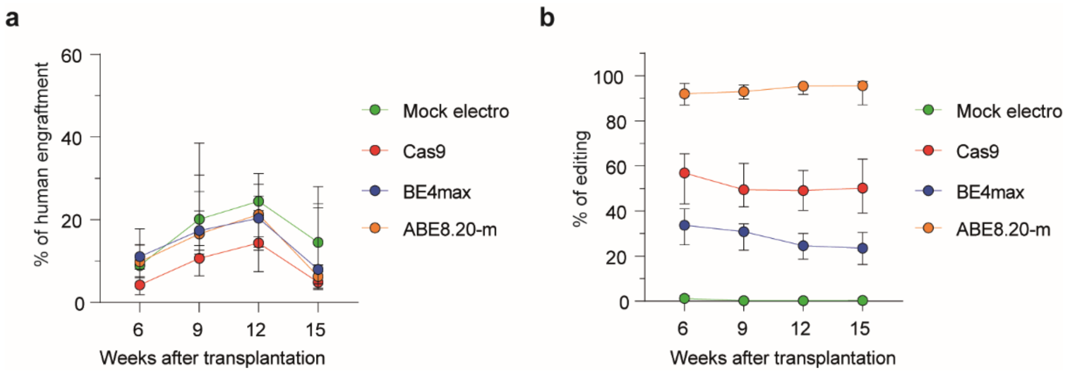

Supplementary Fig. 1. Percentage of human engraftment and editing of treated cells in xenotransplant experiments. **a,b,** Percentage of human cells engraftment (**a**) and editing of treated cells within human graft (**b**) in mice transplanted with CB/mPB HSPCs after B2M exon 2/AAVS1 editing at day 3 post-thawing from Fig. 3b (right), 3g, Extended Data Fig. 2g, 2n for (a) and from Fig. 3c (right), 3h, Extended Data Fig. 2h, 2o (n= 18,18,19,20). Median with IQR. LME followed by post hoc analysis. All statistical tests are two-tailed. n indicate independent animals.

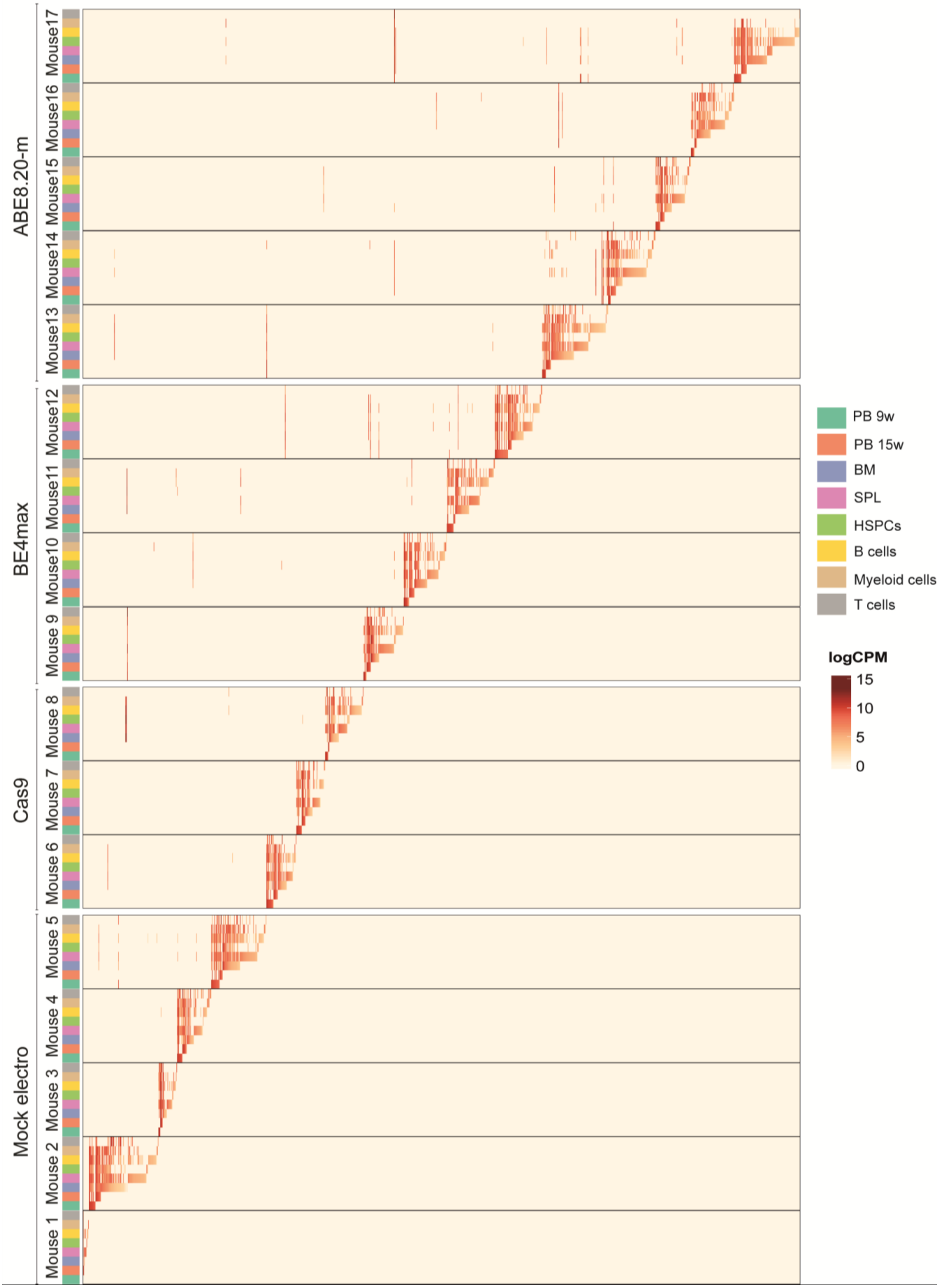

Supplementary Fig. 2. Abundance and inter-/intra-mouse sharing of BARs from clonal tracking analyses. Heatmap of the abundance (red-scaled palette) of BARs (rows) in PB at indicated times

after transplant, hematopoietic organs and lineages (columns) in mice from Fig. 3i. All statistical tests are two-tailed. n indicate independent animals.

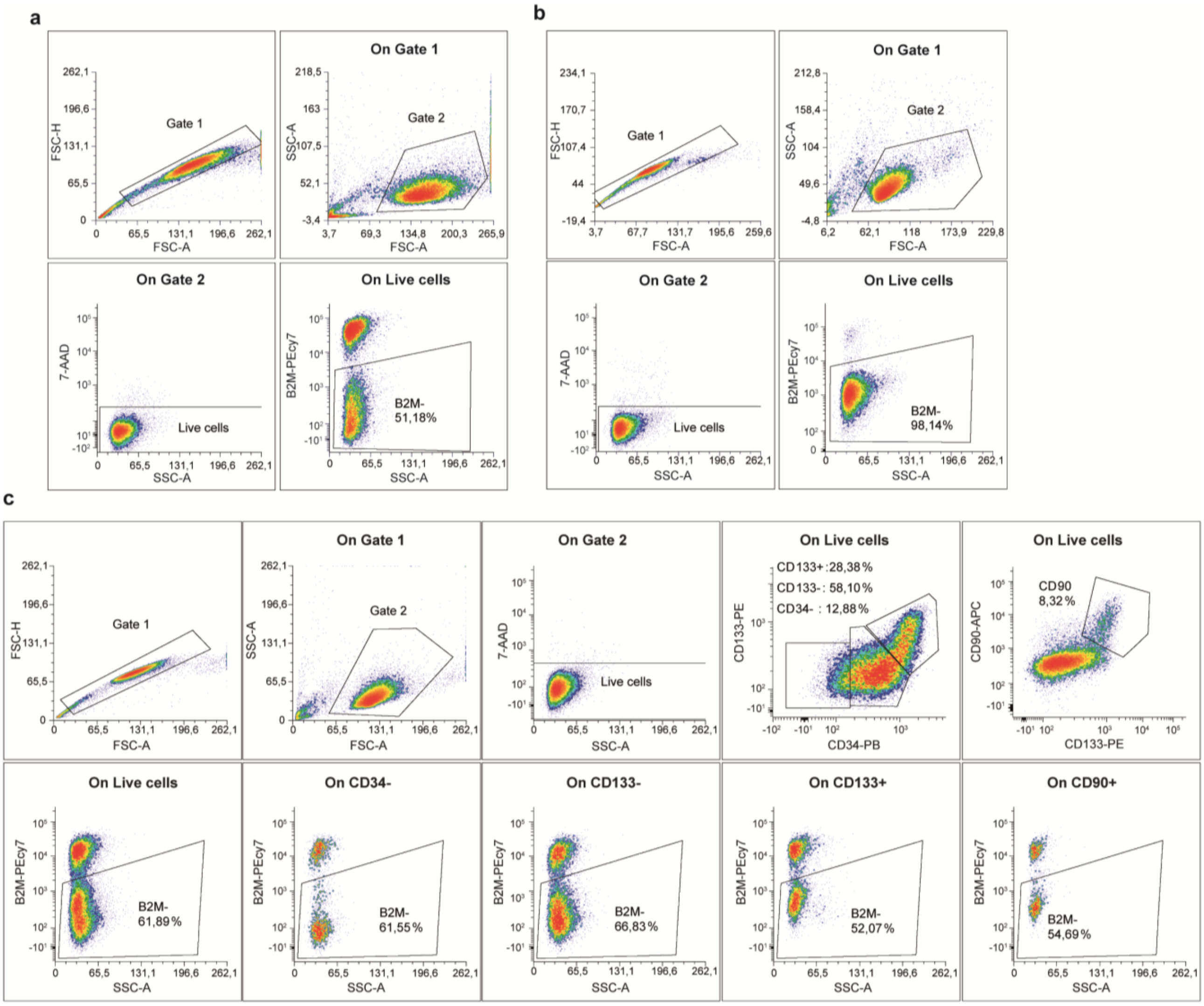

Supplementary Fig. 3. Gating strategies for flow cytometry analyses used for in vitro samples. Gating strategies for the analysis of: **a**, B-lymphoblastoid cells at 7 days after treatments; **b**, Human T cells at 7 days after treatments; **c**, Human HSPCs (CB-/mPB-derived) at 7 days after treatments.

a

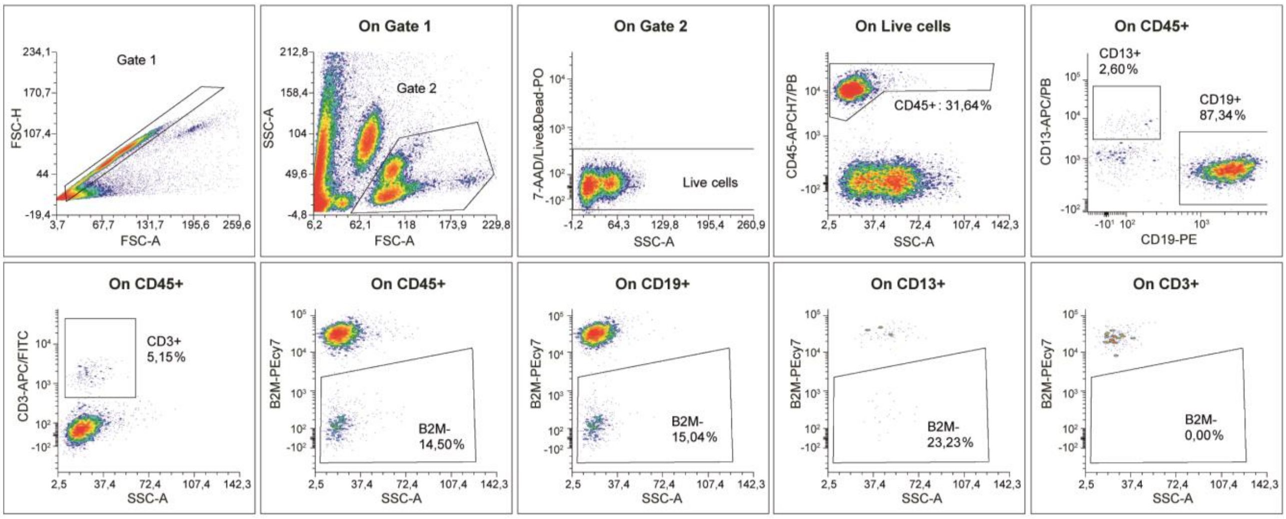

b

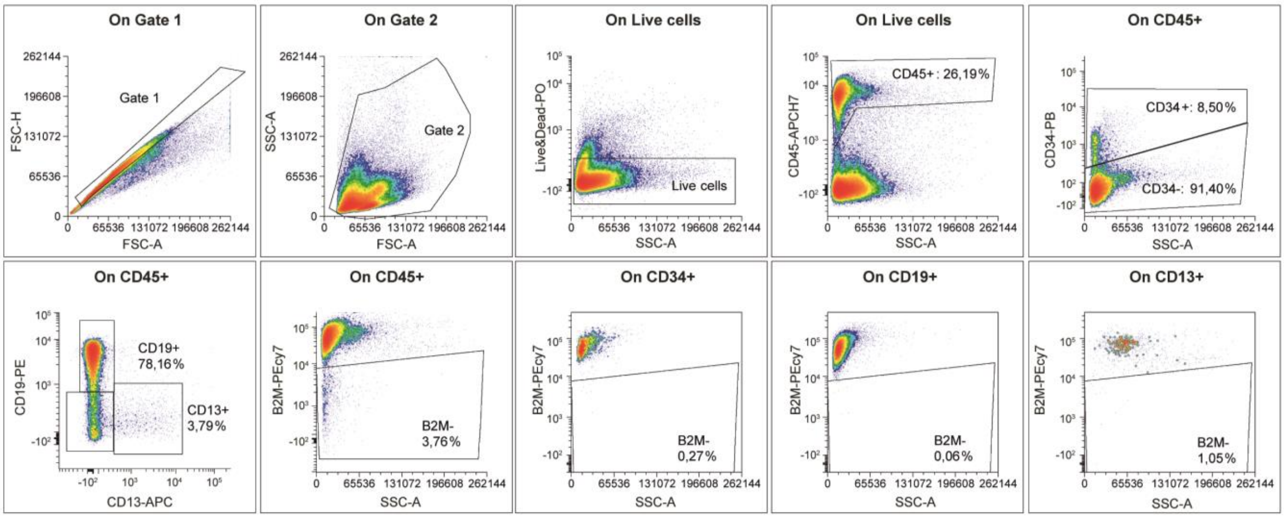

c

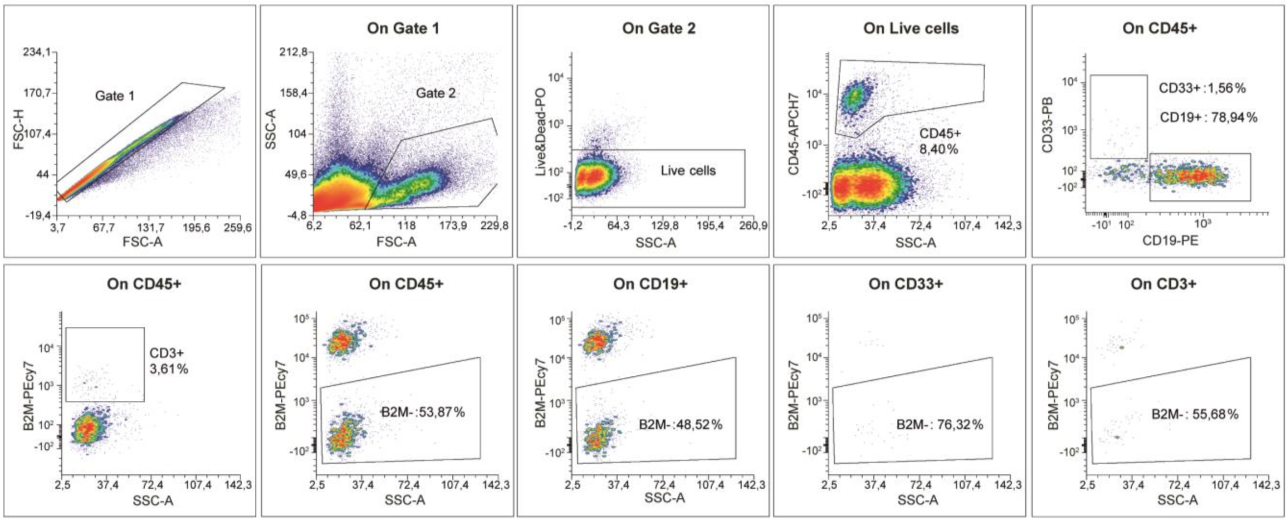

Supplementary Fig. 4. Gating strategies for flow cytometry analyses used for PB, BM and SPL of mice. Gating strategies for the analysis of: **a**, human cells in PB of transplanted NSG mice; **b**,

human cells within BM of transplanted NSG mice at the end of the experiment; **c**, human cells within SPL of transplanted NSG mice at the end of the experiment.
