# Supplementary material for: Genotoxic effects of base and prime editing in human hematopoietic stem cells
Source: Nat Biotechnol. 2023 Sep 7;42(6):877–91. doi: 10.1038/s41587-023-01915-4 (PMC11180610; doi:10.1038/s41587-023-01915-4)
Supplement: Supplementary file 2 — Reporting Summary [file 41587_2023_1915_MOESM2_ESM.pdf]

nature research

Corresponding author(s): Luigi Naldini, Samuele Ferrari

Last updated by author(s): Jul 14, 2023

Reporting Summary

Nature Research wishes to improve the reproducibility of the work that we publish. This form provides structure for consistency and transparency in reporting. For further information on Nature Research policies, see Authors & Referees and the Editorial Policy Checklist.

Statistics

For all statistical analyses, confirm that the following items are present in the figure legend, table legend, main text, or Methods section.

n/a	Confirmed	
☐	☒	The exact sample size (n) for each experimental group/condition, given as a discrete number and unit of measurement
☐	☒	A statement on whether measurements were taken from distinct samples or whether the same sample was measured repeatedly
☐	☒	The statistical test(s) used AND whether they are one- or two-sided<br>*Only common tests should be described solely by name; describe more complex techniques in the Methods section.*
☐	☒	A description of all covariates tested
☐	☒	A description of any assumptions or corrections, such as tests of normality and adjustment for multiple comparisons
☐	☒	A full description of the statistical parameters including central tendency (e.g. means) or other basic estimates (e.g. regression coefficient) AND variation (e.g. standard deviation) or associated estimates of uncertainty (e.g. confidence intervals)
☐	☒	For null hypothesis testing, the test statistic (e.g. F, t, r) with confidence intervals, effect sizes, degrees of freedom and P value noted<br>*Give P values as exact values whenever suitable.*
☒	☐	For Bayesian analysis, information on the choice of priors and Markov chain Monte Carlo settings
☐	☒	For hierarchical and complex designs, identification of the appropriate level for tests and full reporting of outcomes
☐	☒	Estimates of effect sizes (e.g. Cohen's d, Pearson's r), indicating how they were calculated

Our web collection on statistics for biologists contains articles on many of the points above.

Software and code

Policy information about availability of computer code

Data collection	Immunophenotypic analyses were performed on FACS Canto II (BD Pharmingen) using BDFACS Diva software v8.0.1. DNA amplicons were resolved by capillary electrophoresis on 4200 TapeStation System (Agilent). Digital droplet PCR was performed using the QX200 Droplet Digital PCR System (Bio-Rad). High-throughput sequencing was performed by the San Raffaele Center for Omic Sciences (COSR) or Genewiz (Azenta Life Sciences).<br>High-throughput sequencing was performed by Genewiz (Azenta Life Sciences).
Data analysis	Flow cytometry data were analyzed with FCS Express 7 Flow. Digital droplet PCR data were analyzed with QuantaSoftTM Software v1.7.4 (Bio-Rad).<br><br>For target deep sequencing data, for each sample, input reads were trimmed (CRISPResso2 options: –trim_sequences –trimmomatic_command trimmomatic –trimmomatic_options_string 'ILLUMINACLIP:TruSeq3-PE-2.fa:2:30:10 MINLEN:100') to get rid of low-quality positions (score < 30) and to remove Illumina adapters, keeping only trimmed sequences longer than 100 bp to ensure the full coverage of the region of interest. Then, sequences were mapped to the input amplicon reference and the quantification window was set to 1bp around the cut site, as identified by providing the gRNA sequence. Computed alleles were quantified by measuring the number of reads and their relative abundance based on total read counts. Moreover, depending on the type of experiment (i.e., base or prime editing), different input options were given to CRISPResso2 to perform the specific analyses. For base editing analyses, both the targeted and the edited nucleotide were provided (CRISPResso2 options: --base_editor_output --conversion_nuc_from T/G --conversion_nuc_to C/A) in order to measure the frequency of the expected nucleotide substitutions for the specific BE. For the prime editing analyses, the sequences for the pegRNA spacer, extension and scaffold, as well as for the additional nicking gRNA and the reference amplicon, were provided as input to identify and quantify precise prime editing (i.e., carrying only the expected edit), imprecise prime editing (i.e., containing the prime editing and additional modifications, such as partial scaffold-incorporation and indels) and other events. Finally, CRISPResso2 output alleles were post-processed by correcting all the mismatch positions outside the quantification window and re-quantifying the total read counts and consequently the corresponding relative abundances.<br><br>For clonal tracking data, sequencing data were analyzed with the BAR-Seq2 pipeline pipeline (https://bitbucket.org/bereste/bar-seq2). In

details, input reads were pre-processed to trim low-quality bases and keeping sequences having length ≥50bp (options: -m 50 -q 30) to ensure the proper amplicon structure within each read. BARs were then extracted using TagDust and corrected using a community-based strategy on a graph built on the sequence similarity (edit distance ≤ 2). Resulting BARs were quantified based on their abundance (number of supporting reads) and filtered, keeping only those having minimum count equal to 2. The number of clones were calculated for each sample by normalizing the number of unique BARs by the sample VCN.

For RNA-seq data, pre-processing of the input sequences was done with FastQC (v0.11.6) to assess reads quality and trimmomatic to get rid of low-quality sequences. Then, reads were aligned to the human genome assembly (GRCh38) using the STAR software (v2.7.6a) with standard parameters, and abundancies were calculated using the Subread featureCounts function (v2.0.1). Differential Gene Expression (DGE) analysis was performed using the R/Bioconductor package DESeq2 (v1.30.0), normalizing for library size using DESeq2's median of ratios. p-values were corrected using false discovery rate (FDR), and genes having FDR < 0.05 were considered as differentially expressed. Post-analyses on DGE results were performed with the R/Bioconductor package ClusterProfiler (v4.7.1), using the Hallmark collection from MSigDB as reference database. Visualization of the (spliced) alignments on the TP73 gene was done with Integrative Genomes Viewer (IGV v2.8.0).
Variant calling on RNA-Seq base editing data was performed exploiting different tools similarly to [56,57]. In details, reads from replicates of each condition were pulled together, down sampled to 120M, and aligned to the human genome assembly (GRCh38) using the STAR (v2.7.6a). Then, following the GATK "Best Practice Workflows" as reported in [20], duplicates were marked using Picard (v2.25.6) MarkDuplicates and GATK (v4.2.0) SplitNCigarReads was used to split reads containing Ns. Variants were then called using three different tools, namely, HaplotypeCaller (with options --min-base-quality-score 20, --dont-use-soft-clipped-bases, and –standard-min-confidence-threshold-for-calling 20), Mutect2 (in tumor-only mode, with options --disable-read-filter MateOnSameContigOrNoMappedMateReadFilter), and FreeBayes (v1.3.5). Nucleotide composition of each position was also assessed using REDItools (https://github.com/tflati/reditools2.0) on each sample, discarding all the positions having coverage lower than 20 and base quality lower than 30 to avoid errors due to low sampling. Next, variants called by each tool in the untreated controls were filtered out in the treated samples to enrich for private mutations. This procedure retained only variants in high-quality genomic positions in both treated and untreated sample, for which the untreated sample showed ≥ 99% of reads supporting the reference, non-mutant, base at the position of the mutation (based on REDItools). The final lists of variants for each sample were made by those called by all tools and passing the filtering procedure (intersection).

All WES were performed by Genewiz (Azenta) using the Agilent SureSelect Human All Exon V7 kit and running an Illumina NovaSeq 2x150bp with an estimated output of ~50Gb (500X) or ~10Gb (100X) per sample. WES data were analyzed following the GATK "Best Practice Workflows" to identify variants in each sample. Briefly, the quality of the input reads was assessed using FastQC (v0.11.9), also trimming low-quality bases using trim-galore (v0.6.6), and finally, for samples retrieved from mice, a disambiguation was preformed to get rid of possible mouse contaminations. The latter operation was performed as described in [58], i.e., by aligning sequences to human and mouse reference genomes, and then assigning each read to the organism showing the best alignment. Next, most abundant samples were randomly downsampled to 300M reads, 230M reads or 50M reads according to the experiment using the Seqtk toolkit (v1.3) to avoid sample unbalance. Reads were then aligned to the human genome assembly (GRCh38) using the BWA (v0.7.17). Alignments were processed to mark duplicates using Picard (v2.25.6) MarkDuplicates, and GATK (v4.2.0) BaseRecalibrator + ApplyBQSR were used to recalibrate base quality scores on dbSNP known sites. HaplotypeCaller in GVCF mode was used to call variants in each sample, which were then combined using CombineGVCFs and genotyped with GenotypeGVCFs. Resulting variants were filtered using VariantFiltration based on their 'QualityByDepth' (i.e., --filter-expression 'QD < 2.0') and overall coverage 'DP' (i.e., --filter-expression 'DP < 50'). To identify private variants belonging to each sample additional filters were applied, i.e., variants having low genotype quality (i.e., GQ < 80) and low coverage (i.e., DP < 100 stringent and DP < 10 relaxed, respectively) were removed. The "Mock electro" in vitro sample for each experiment was used as germline reference, and its variants were filtered out from all other samples, as they were considered as present in the initial cell population and not induced by treatments. Moreover, for the BE4max group in WES in Fig. 4, variants of the samples positively sorted for the B2M editing were merged with that of the negative samples, for each mouse. A final refinement was performed to get rid of multi-allelic variants (mainly involving repetitive sequences). Remaining variants were annotated using SnpEff (v5.0) on the canonical isoform from the GRCh38.p13.RefSeq reference database. Downstream analysis of the final variants was done by classifying them based on their type (insertion, deletion, or SNV), and focusing on all SNV to classify mutation events. Assessment of variants using a panel of cancer related genes was performed based on variant annotations. An additional focus on low-frequency variants was performed for WES in Fig. 5q-s by using Mutect2 to call variants and then filtering those having coverage lower than 10. To enrich for variants private of each colony, including those installed by the treatment, we kept for the analysis only those being in the expected range of variant allele frequency (i.e., between 0.05 and 0.2), considering that each pool was composed by six colonies (12 alleles).

For manuscripts utilizing custom algorithms or software that are central to the research but not yet described in published literature, software must be made available to editors/reviewers. We strongly encourage code deposition in a community repository (e.g. GitHub). See the Nature Research guidelines for submitting code & software for further information.

Data

Policy information about availability of data

All manuscripts must include a data availability statement. This statement should provide the following information, where applicable:

- Accession codes, unique identifiers, or web links for publicly available datasets
- A list of figures that have associated raw data
- A description of any restrictions on data availability

All relevant data are included in the manuscript. The reagents described in this manuscript are available under a material transfer agreement with IRCCS Ospedale San Raffaele and Fondazione Telethon; requests for materials should be addressed to S.F. and L.N. BAR-Seq, RNA-Seq, and targeted deep sequencing data are deposited at GEO (accession number: GSE218464)[61], while WES data are deposited at ENA with the following accession numbers: PRJEB58344 (in vivo experiment with standard mRNA constructs) [62], PRJEB64207 (experiment on HSPC derived colonies)[63], and PRJEB64407 (in vivo experiment with standard and optimized mRNA constructs) [64].
All other raw data from main figures have been deposited at Mendeley and are publicly available as of the date of publication [65].
The scripts for all bioinformatic analyses of RNA-seq, WES, BAR-Seq, and deep sequencing of the locus are freely available at http://www.bioinfotiget.it/gitlab/custom/fiumara_baseprimeed2022 [66]

Field-specific reporting

Please select the one below that is the best fit for your research. If you are not sure, read the appropriate sections before making your selection.

☒ Life sciences ☐ Behavioural & social sciences ☐ Ecological, evolutionary & environmental sciences

For a reference copy of the document with all sections, see nature.com/documents/nr-reporting-summary-flat.pdf

Life sciences study design

All studies must disclose on these points even when the disclosure is negative.

Sample size	Sample size for each experiment was determined by the total number of available treated cells, which is constrained by the human source of the material, to be split among each experimental conditions. Whenever possible we aimed to reach at least 5 replicates per group, thus reaching a minimum and sensible operational criterial for carrying out at least nonparametric statistics. In some in vivo experiments, such as secondary transplantation, the total number of available cells was more constrained and limited to what could be harvested from the primary recipients.
Data exclusions	For in vivo experiments failure during injection of treated HSPCs, confirmed by graft failure, in recipient animals led to exclusion of that mouse from the experimental group. No other data or sample were excluded from analysis. All these criteria were pre-established.
Replication	All experiments (except for some experiments reported in Fig.1c, m; Fig. 3h-o; Extended Data Fig. 1a,b,e; Extended Data Fig. 3i-l; Extended Data Fig. 5l,m) were repeated three or more times. Number of biological replicates is specified for each experiment in figure legends. All attempts at replication were successful. Inferential techniques were applied in presence of adequate sample sizes ($n \geq 5$), otherwise only descriptive statistics are reported.
Randomization	For tissue culture experiments, conditions were assigned to wells in 96- or 48- or 24-well plates; plate position is not expected to affect editing efficiency. Mice were randomly distributed to each experimental group. For WES in Fig4, 2 or 3 mice/group from the clonal tracking experiment in Fig. 2i were selected randomly; For WES in Fig5 3 mice per group were selected randomly.
Blinding	Experiments were not performed in blind-fashion. Sequencing data were analyzed by a blinded operator by using and automated scripts with limited experimenter intervention. Blood samples ware collected by a blind operator and mice were identified by a code not reflecting the treatment group.

Reporting for specific materials, systems and methods

We require information from authors about some types of materials, experimental systems and methods used in many studies. Here, indicate whether each material, system or method listed is relevant to your study. If you are not sure if a list item applies to your research, read the appropriate section before selecting a response.

Materials & experimental systems

n/a	Involved in the study	
☐	☒	Antibodies
☐	☒	Eukaryotic cell lines
☒	☐	Palaeontology
☐	☒	Animals and other organisms
☐	☒	Human research participants
☒	☐	Clinical data

Methods

n/a	Involved in the study	
☒	☐	ChIP-seq
☐	☒	Flow cytometry
☒	☐	MRI-based neuroimaging

Antibodies

Antibodies used

*CD34-VioBlue, anti-human (Supplier: Miltenyi Biotec; Catalog n°130-113-182; Clone: AC136; Dilution: 1:50)
*CD133/2-PE, anti-human (Supplier: Miltenyi Biotec; Catalog n°130-112-157; Clone: REA816; Dilution: 1:50)
*CD90-APC, anti-human (Supplier: BD Biosciences; Catalog n°559869; Clone: 5E10; Dilution: 1:33)
*B2M-Pecy7, anti-human (Supplier: Biolegend; Catalog n°316318; Clone: 2M2; Dilution: 1:100)
*CD45-VioBlue, anti-human (Supplier: BioLegend; Catalog n°304029; Clone: HI30; Dilution: 1:50)
*CD45-APC, anti-human (Supplier: BD Biosciences; Catalog n°340910; Clone: 2D1; Dilution: 1:50)
*CD45-APC-Vio770, anti-human (Supplier: BD Biosciences; Catalog n°348815; Clone: 2D1; Dilution: 1:50)
*CD19-PE, anti-human (Supplier: BD Biosciences; Catalog n°345789; Clone: SJ25C1; Dilution: 1:50)
*CD3-APC, anti-human (Supplier: BD Biosciences; Catalog n° 555335; Clone: UCHT1; Dilution: 1:50)
*CD3-FITC, anti-human (Supplier: BD Biosciences; Catalog n° 345763; Clone: SK7; Dilution: 1:50)
*CD13-APC, anti-human (Supplier: BD Biosciences; Catalog n°557454; Clone: WM15; Dilution: 1:50)
*CD13- BV421, anti-human (Supplier: BD Biosciences; Catalog n°562596; Clone WM15
*CD33-PE-Vio770, anti-human (Supplier: Miltenyi Biotec; Catalog n°130-113-350; Clone: WM15; Dilution: 1:50)
*CD38-PerCP/Cyanine5.5, anti-human (Supplier: BioLegend; Catalog n°356614; Clone: HB-7; Dilution: 1:20)

*CD271 (NGFR)-PE, anti-human (Supplier: Miltenyi Biotec; Catalog n°130-113-421; Clone: ME20.4-1.H4; Dilution: 1:50)
*CD271 (NGFR)-APC, anti-human (Supplier: Miltenyi Biotec; Catalog n°130-113-418; Clone: ME20.4-1.H4; Dilution: 1:50)
*human Fc blocking (Supplier: Supplier: Miltenyi Biotec; Catalog n°130-092-283; Dilution: 1:50)
*mouse Fc blocking (Supplier: BD Biosciences; Catalog n°553142; Dilution: 1:100)

Validation

https://www.miltenyibiotec.com/upload/assets/dataSheet_p42699_eng_GBR.pdf
https://www.miltenyibiotec.com/upload/assets/dataSheet_p41491_eng_GBR.pdf
https://www.bdbiosciences.com/content/bdb/paths/generate-tds-document.us.561971.pdf
https://d1spbj2x7qk4bg.cloudfront.net/en-ie/products/pe-cyanine7-anti-human-beta2-microglobulin-antibody-13839?pdf=true&displayInline=true&leftRightMargin=15&topBottomMargin=15&filename=PE/Cyanine7%20anti-human%20β2-microglobulin%20Antibody.pdf&v=20220818063102
https://d1spbj2x7qk4bg.cloudfront.net/nl-be/products/pacific-blue-anti-human-cd45-antibody-3331?pdf=true&displayInline=true&leftRightMargin=15&topBottomMargin=15&filename=Pacific%20Blue™%20anti-human%20CD45%20Antibody.pdf&v=20220831123135
https://www.bdbiosciences.com/content/dam/bdb/products/global/reagents/flow-cytometry-reagents/clinical-diagnostics/single-color-antibodies-asr-ivd-ce-ivd/655873_base/pdf/23-5097.pdf
https://www.bdbiosciences.com/content/dam/bdb/products/global/reagents/flow-cytometry-reagents/clinical-diagnostics/single-color-antibodies-asr-ivd-ce-ivd/655873_base/pdf/23-5097.pdf
https://www.bdbiosciences.com/content/bdb/paths/generate-tds-document.kr.340364.pdf
https://www.bdbiosciences.com/content/bdb/paths/generate-tds-document.us.555335.pdf
https://www.bdbiosciences.com/content/dam/bdb/products/global/reagents/flow-cytometry-reagents/clinical-diagnostics/single-color-antibodies-asr-ivd-ce-ivd/345766_base/pdf/23-5019.pdf
https://www.bdbiosciences.com/content/bdb/paths/generate-tds-document.us.557454.pdf
https://www.bdbiosciences.com/content/dam/bdb/products/global/reagents/flow-cytometry-reagents/research-reagents/single-color-antibodies-ruo/562596_base/pdf/562596.pdf
https://www.miltenyibiotec.com/_Resources/Persistent/e280a589ae1279e1b469872569d2f53a64e88129/DS_CD33_Antibody_anti-human_PE-Vio®_770_AC104.3E3_130-113-350.pdf
https://d1spbj2x7qk4bg.cloudfront.net/en-us/products/percp-cyanine5-5-anti-human-cd38-antibody-8608?pdf=true&displayInline=true&leftRightMargin=15&topBottomMargin=15&filename=PerCP/Cyanine5.5%20anti-human%20CD38%20Antibody.pdf&v=20220606063221
https://www.miltenyibiotec.com/_Resources/Persistent/94704693cc7ec1d7591473b844f13c736fa66054/DS_CD271_LNGFR_Antibody_anti-human_PE_ME20.4-1.H4_130-113-421.pdf
https://www.miltenyibiotec.com/_Resources/Persistent/15881e147bb47891db3851d656e7a14e8ac9ddf8/DS_CD271_LNGFR_Antibody_anti-human_APC_ME20.4-1.H4_130-113-418.pdf
https://www.miltenyibiotec.com/_Resources/Persistent/93b47706211e224f68c262365dfb7eb8e3fb8098/DS_FcR%20Blocking%20Reagent_h.pdf
https://www.bdbiosciences.com/content/dam/bdb/products/global/reagents/flow-cytometry-reagents/research-reagents/single-color-antibodies-ruo/553141_base/pdf/553142.pdf

Eukaryotic cell lines

Policy information about cell lines

Cell line source(s): K-562 (ATCC), B-lymphoblastoid (established in the lab)

Authentication: K-562 and B-lymphoblastoid Cells were grown from a working cell bank established in the laboratory. Cell line authentication was not performed

Mycoplasma contamination: Test for mycoplasma contamination was negative.

Commonly misidentified lines (See ICLAC register): No commonly misidentified lines were used in the study

Animals and other organisms

Policy information about studies involving animals; ARRIVE guidelines recommended for reporting animal research

Laboratory animals: NOD-SCID-IL2Rg-/- (NSG) female mice (7-10 weeks of age) were obtained from Jackson Laboratory. Animals were maintained in Specific pathogen-free (SPF) animal research facilities with a 12h/12h dark/light cycle and standardized temperature (22 +/- 2°C) and humidity (55 +/- 5%).

Wild animals: The study did not involve wild animals.

Field-collected samples: The study did not involve samples collected from the field.

Ethics oversight: All experiments and procedures involving animals were performed with the approval of the Animal Care and Use Committee of the San Raffaele Hospital (IACUC: #1206) and authorized by the Italian Ministry of Health and local authorities accordingly to Italian law.

Note that full information on the approval of the study protocol must also be provided in the manuscript.

Human research participants

Policy information about studies involving human research participants

Population characteristics	Healthy donors were used as T cells and HSPC sources. T and mobilized peripheral blood HSPC donors range from 18 to 50 years old and were equally disrtibuted among males and females. Genotypic information, past and current diagnosis and treatment categories are not known as they were not relevant for the study design and donors were anonimized.
Recruitment	Primary T cells were isolated from buffy coats from healthy donors as results of residues of blood product donations. CB HSPCs were purchased from Lonza, mPB HSPCs were purified from Mobilized Leukopak (AllCells).
Ethics oversight	Buffy coats were obtained in accordance with the Declaration of Helsinki, as anonymized residues of blood donations, used upon signature of specific institutional informed consent for blood product donation by healthy blood donors. HSPCs were obtained in accordance with the Declaration of Helsinki. T and HSPCs collection were approved by the Ospedale San Raffaele Scientific Institute bioethical committee (TIGET-09 and TIGET-HPCT).

Note that full information on the approval of the study protocol must also be provided in the manuscript.

Flow Cytometry

Plots

Confirm that:

☒ The axis labels state the marker and fluorochrome used (e.g. CD4-FITC).

☒ The axis scales are clearly visible. Include numbers along axes only for bottom left plot of group (a 'group' is an analysis of identical markers).

☒ All plots are contour plots with outliers or pseudocolor plots.

☒ A numerical value for number of cells or percentage (with statistics) is provided.

Methodology

Sample preparation	From 5.0x10e4 to 2.0x10e5 cells either from culture or mouse-derived samples were analyzed. Cells were stained for 15' at 4°C with antibodies listed in Supplementary Table 4 in a final volume of 100µl and then washed with DPBS+2% heat-inactivated FBS. Single stained and fluorescence-minus-one-stained cells were used as controls. The Live/Dead Fixable Dead Cell Stain Kit (Thermo Fisher) or 7-aminoactinomycin D (Sigma Aldrich) were included during sample preparation according to the manufacturer's instructions to identify dead cells.
Instrument	Immunophenotypic analyses were performed on FACS Canto II (BD Pharmingen). Cell sorting was performed on BD FACSAria Fusion (BD Biosciences)
Software	The flow cytometry data were analyzed with FCS Express 7 Flow.
Cell population abundance	The abundance of population within post-sort fractions was >1,000 cells for clonal tracking experiment and >500,000 for WES experiments. The purity of the sorted samples (>95%) was determined by re-running them by flow cytometry.
Gating strategy	The gating strategies are provided in Supplementary Figure 3 and Supplementary Figure 4. Hierarchical gating was performed as follow:<br>In vitro experiment on CD34 cells: Singlets (FSC-A/FSC-H), Physical parameters (FSC-A/SSC-A), live cells (7AAD negative), cell composition (hCD34, hCD133 cells, hCD90 cells), edited cells (B2M-).<br>Blood samples: Singlets (FSC-A/FSC-H), Physical parameters (FSC-A/SSC-A), live cells (7AAD negative), human cells (hCD45), human cell composition (CD19,CD13,CD3), edited cells (B2M-).<br>Bone marrow samples: Singlets (FSC-A/FSC-H), Physical parameters (FSC-A/SSC-A), live cells (Live/Dead negative), human cells (hCD45), human cell composition (CD19,CD13,CD34), edited cells (B2M-).<br>Spleen sample: Singlets (FSC-A/FSC-H), Physical parameters (FSC-A/SSC-A), live cells (Live/Dead negative), human cells (hCD45), human cell composition (CD19,CD13,CD34), edited cells (B2M-).<br>Boundaries were defined using negative and positive samples.

☒ Tick this box to confirm that a figure exemplifying the gating strategy is provided in the Supplementary Information.
