# Supplementary material for: Genotoxic effects of base and prime editing in human hematopoietic stem cells
Source: Nat Biotechnol. 2023 Sep 7;42(6):877–91. doi: 10.1038/s41587-023-01915-4 (PMC11180610; doi:10.1038/s41587-023-01915-4)

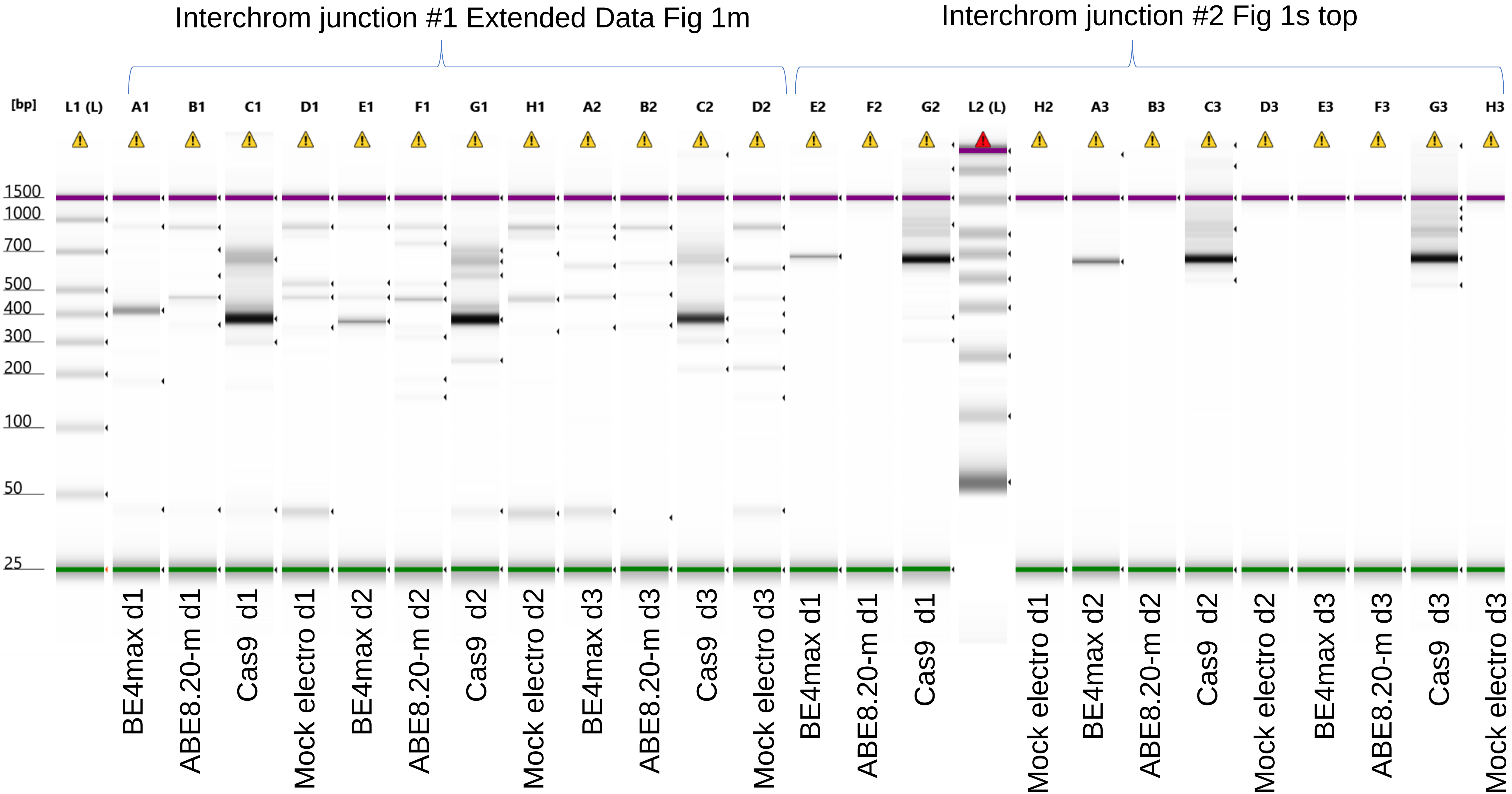

Interchrom junction #1 Extended Data Fig 1m
Interchrom junction #2 Fig 1s top
[bp]
L1 (L)
A1
B1
C1
D1
E1
F1
G1
H1
A2
B2
C2
D2
E2
F2
G2
L2 (L)
H2
A3
B3
C3
D3
E3
F3
G3
H3
1500
1000
700
500
400
300
200
100
50
25
BE4max d1
ABE8.20-m d1
Cas9 d1
Mock electro d1
BE4max d2
ABE8.20-m d2
Cas9 d2
Mock electro d2
BE4max d3
ABE8.20-m d3
Cas9 d3
Mock electro d3
BE4max d1
ABE8.20-m d1
Cas9 d1
Mock electro d1
BE4max d2
ABE8.20-m d2
Cas9 d2
Mock electro d2
BE4max d3
ABE8.20-m d3
Cas9 d3
Mock electro d3

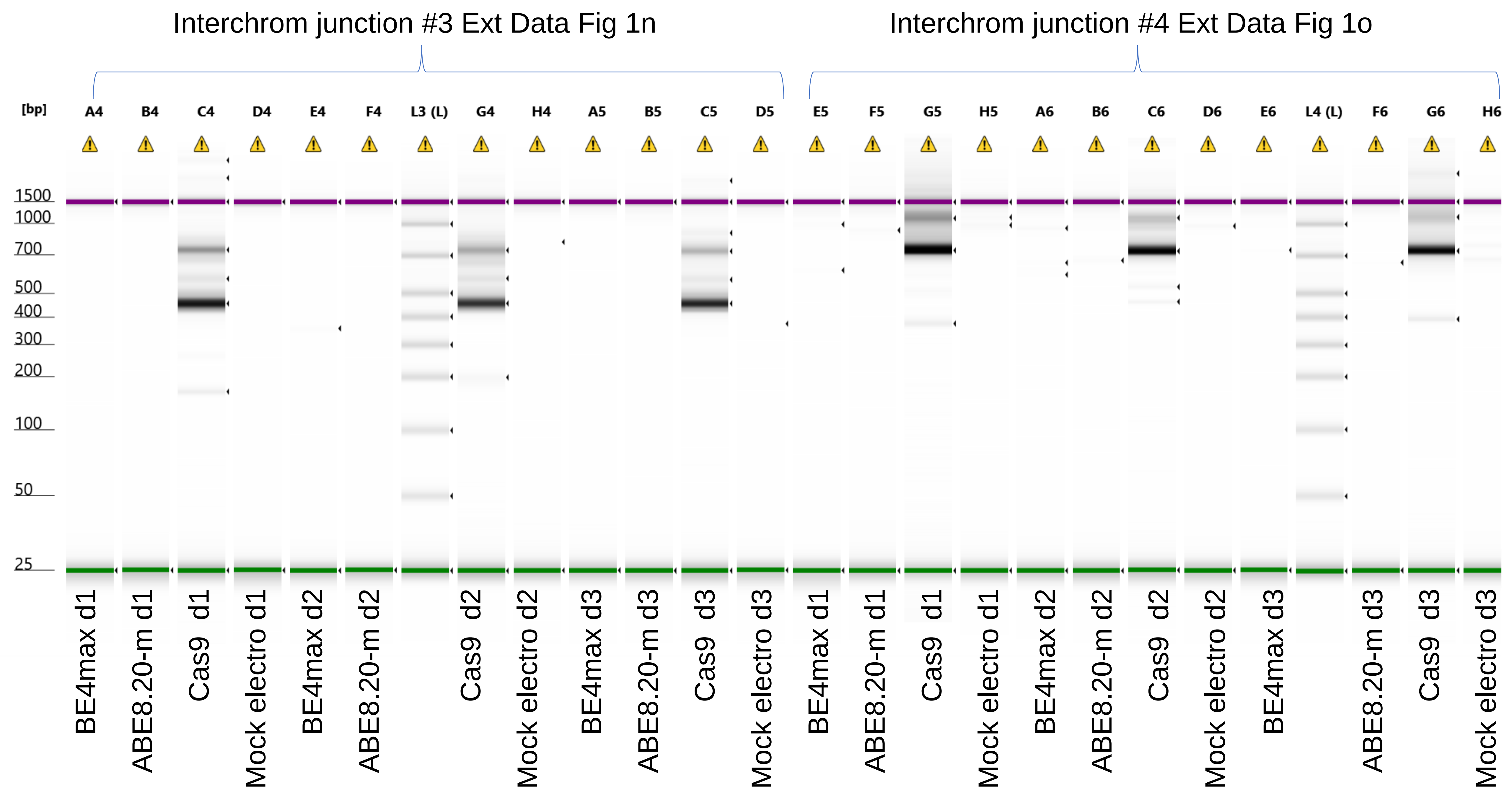
Interchrom junction #3 Ext Data Fig 1n
Interchrom junction #4 Ext Data Fig 1o
[bp]
A4
B4
C4
D4
E4
F4
L3 (L)
G4
H4
A5
B5
C5
D5
E5
F5
G5
H5
A6
B6
C6
D6
E6
L4 (L)
F6
G6
H6
1500
1000
700
500
400
300
200
100
50
25
BE4max d1
ABE8.20-m d1
Cas9 d1
Mock electro d1
BE4max d2
ABE8.20-m d2
Cas9 d2
Mock electro d2
BE4max d3
ABE8.20-m d3
Cas9 d3
Mock electro d3
BE4max d1
ABE8.20-m d1
Cas9 d1
Mock electro d1
BE4max d2
ABE8.20-m d2
Cas9 d2
Mock electro d2
BE4max d3
ABE8.20-m d3
Cas9 d3
Mock electro d3

Interchrom junction #2 Fig 1s bottom (std); Fig 5i (opt)

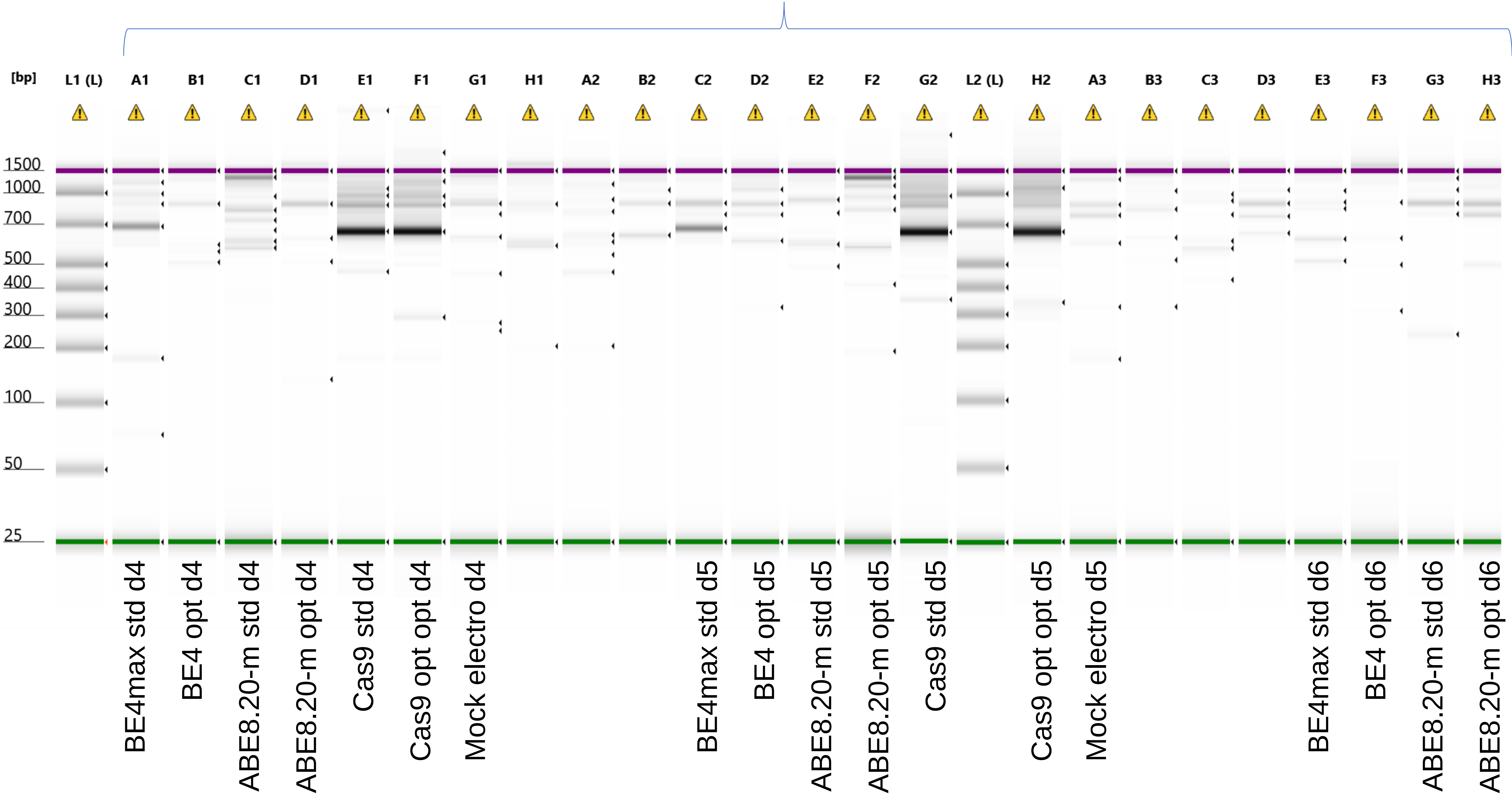

Interchrom junction #2 Fig 1s bottom (std); Fig 5i (opt)

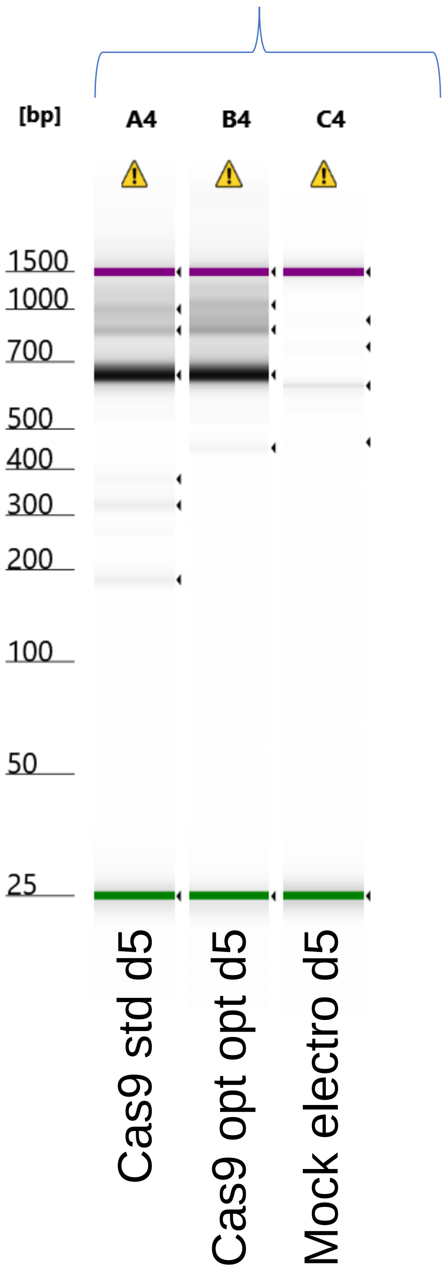

Extended Data figure 4e

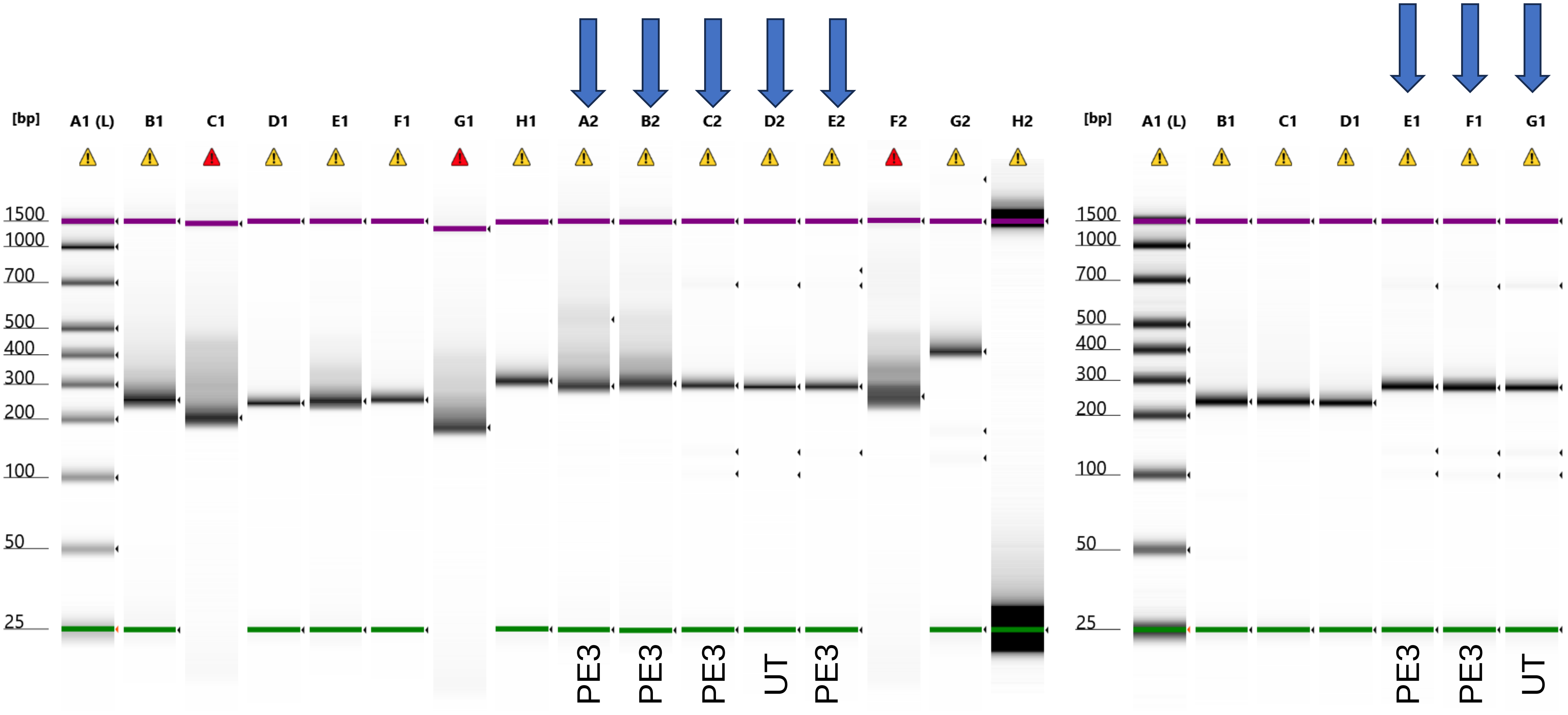

Supplement: Supplementary file 4 — Uncropped scans of gels. [file 41587_2023_1915_MOESM4_ESM.pdf]
